# The Link between Microbial Diversity and Nitrogen Cycling in Marine Sediments Is Modulated by Macrofaunal Bioturbation

**DOI:** 10.1371/journal.pone.0130116

**Published:** 2015-06-23

**Authors:** Maryam Yazdani Foshtomi, Ulrike Braeckman, Sofie Derycke, Melanie Sapp, Dirk Van Gansbeke, Koen Sabbe, Anne Willems, Magda Vincx, Jan Vanaverbeke

**Affiliations:** 1 Marine Biology Research Group, Biology Department, Ghent University, Ghent, Belgium; 2 CeMoFE, Ghent University, Ghent, Belgium; 3 The Food and Environment Research Agency, Sand Hutton, York, United Kingdom; 4 Laboratory of Protistology and Aquatic Ecology, Department of Biology, Ghent University, Ghent, Belgium; 5 Laboratory of Microbiology, Department of Biochemistry and Microbiology, Ghent University, Ghent, Belgium; CAS, CHINA

## Abstract

**Objectives:**

The marine benthic nitrogen cycle is affected by both the presence and activity of macrofauna and the diversity of N-cycling microbes. However, integrated research simultaneously investigating macrofauna, microbes and N-cycling is lacking. We investigated spatio-temporal patterns in microbial community composition and diversity, macrofaunal abundance and their sediment reworking activity, and N-cycling in seven subtidal stations in the Southern North Sea.

**Spatio-Temporal Patterns of the Microbial Communities:**

Our results indicated that bacteria (total and β-AOB) showed more spatio-temporal variation than archaea (total and AOA) as sedimentation of organic matter and the subsequent changes in the environment had a stronger impact on their community composition and diversity indices in our study area. However, spatio-temporal patterns of total bacterial and β-AOB communities were different and related to the availability of ammonium for the autotrophic β-AOB. Highest bacterial richness and diversity were observed in June at the timing of the phytoplankton bloom deposition, while richness of β-AOB as well as AOA peaked in September. Total archaeal community showed no temporal variation in diversity indices.

**Macrofauna, Microbes and the Benthic N-Cycle:**

Distance based linear models revealed that, independent from the effect of grain size and the quality and quantity of sediment organic matter, nitrification and N-mineralization were affected by respectively the diversity of metabolically active β-AOB and AOA, and the total bacteria, near the sediment-water interface. Separate models demonstrated a significant and independent effect of macrofaunal activities on community composition and richness of total bacteria, and diversity indices of metabolically active AOA. Diversity of β-AOB was significantly affected by macrofaunal abundance. Our results support the link between microbial biodiversity and ecosystem functioning in marine sediments, and provided broad correlative support for the hypothesis that this relationship is modulated by macrofaunal activity. We hypothesized that the latter effect can be explained by their bioturbating and bio-irrigating activities, increasing the spatial complexity of the biogeochemical environment.

## Introduction

Coastal marine sediments play a pivotal role in the ecology of shallow marine ecosystems. They receive up to 30% of the pelagically produced organic matter [1], which is mineralised and returned to the water column as inorganic nutrients [2], further supporting primary and secondary production.

As mineralization is essentially a microbial process, investigations on how microbial diversity affects benthic ecosystem functioning are called for. However, while a positive biodiversity-ecosystem functioning link has now been established for many ecosystems [3,4], little is as yet known about the biodiversity-ecosystem functioning relationship in natural microbial systems (but see [5–7]), with various relationships being reported (positive [8–12], negative [11,13] or non-significant [10–12,14,15]). This biodiversity-ecosystem functioning relationship can be modulated by environmental factors [16] such as sediment nitrogen content, carbon stable isotope ratios and sediment chlorophyll a concentrations [17].

While the quantity and quality of organic matter in coastal sediments, and the intensity of the mineralization is often related to water column processes (i.e. timing and extent of phytoplankton bloom and water temperature [1]), the distribution of the organic matter in the sediment, and factors affecting mineralization are locally affected by the activities of the sediment-inhabiting larger macrofaunal organisms. While foraging for food, these animals rework and irrigate the sediment, transporting organic matter and oxygen to deeper layers, and enhancing the exchange of solutes between the water column and pore waters [18–20]. Hence, the activities of the macrofauna result in additional complexity within the sediment matrix. This affects microbial abundance [21,22], diversity [23] and activity—mineralization, nitrification and denitrification [24–28]. However, the impact of bioturbation on microbial communities was mainly derived from the relation between single species of large burrowing macrofauna (ecosystem engineers) and total bacterial community while less attention was paid to total archaeal community [29] and nitrifying organisms [30,31]. In addition, integrated studies including different sediment types, repeated over time, investigating the link between natural macrofaunal community, microbial communities and rates of ecosystem functioning are not available yet.

In a previous companion paper [26] the effect of local environmental conditions and the presence and activity of macrofauna on the benthic nitrogen cycle was investigated, given the high importance of nitrogen as a key limiting factor for pelagic primary production [32]. However, as it is well-known that these processes are driven by microbial activities, it is of importance to close the existing gap between marine ‘macro-ecologists’ (focusing on the link macrobenthos-ecosystem functioning) and marine ‘micro-ecologists’ (focusing on the link microbial communities-ecosystem functioning). In the present study, we focused on microbiota involved in N-cycling processes. Ammonia oxidization is the first step in nitrification, central to the cycling of nitrogen in the environment and when coupled with denitrification results in loss of nitrogen from marine environments, and can be performed by ammonia-oxidizing bacteria (AOB) and ammonia-oxidizing archaea (AOA) [33]. New methods targeting functional genes encoding enzymes involved in specific N transformations now allow direct identification and quantification of the microorganisms involved in N-cycling. Here, we focused on ammonia-oxidizing Beta-Proteobacteria (β-AOB) and AOA, by specifically targeting characteristic functional genes, respectively the bacterial *amoA* and archaeal *amoA* genes.

The main objective of this study is to investigate *(i)* whether there is a link between microbial diversity and benthic ecosystem functioning, i.c. the N-cycle (nitrification, denitrification and N-mineralization) in shallow subtidal marine sediments; and *(ii)* if it is modulated by macrofaunal density and/or activity. To this end, we first investigated the link between composition and diversity of microbial communities (active β-AOB and AOA as well as total bacteria and archaea) with environmental variables (i.c. sediment grain size and organic matter content and quality) and macrofaunal density and functional diversity. In a second step, we investigated whether nitrification, denitrification and N-mineralization rates were related to the diversity of the microbial communities and whether this was affected by macrofaunal density and functional diversity. We used the Bioturbation Potential of a macrofaunal community (BPc) as proxy for macrobenthic activity [34,35].

## Materials and Methods

### Study site, sampling and experimental set-up

**Fig 1:**
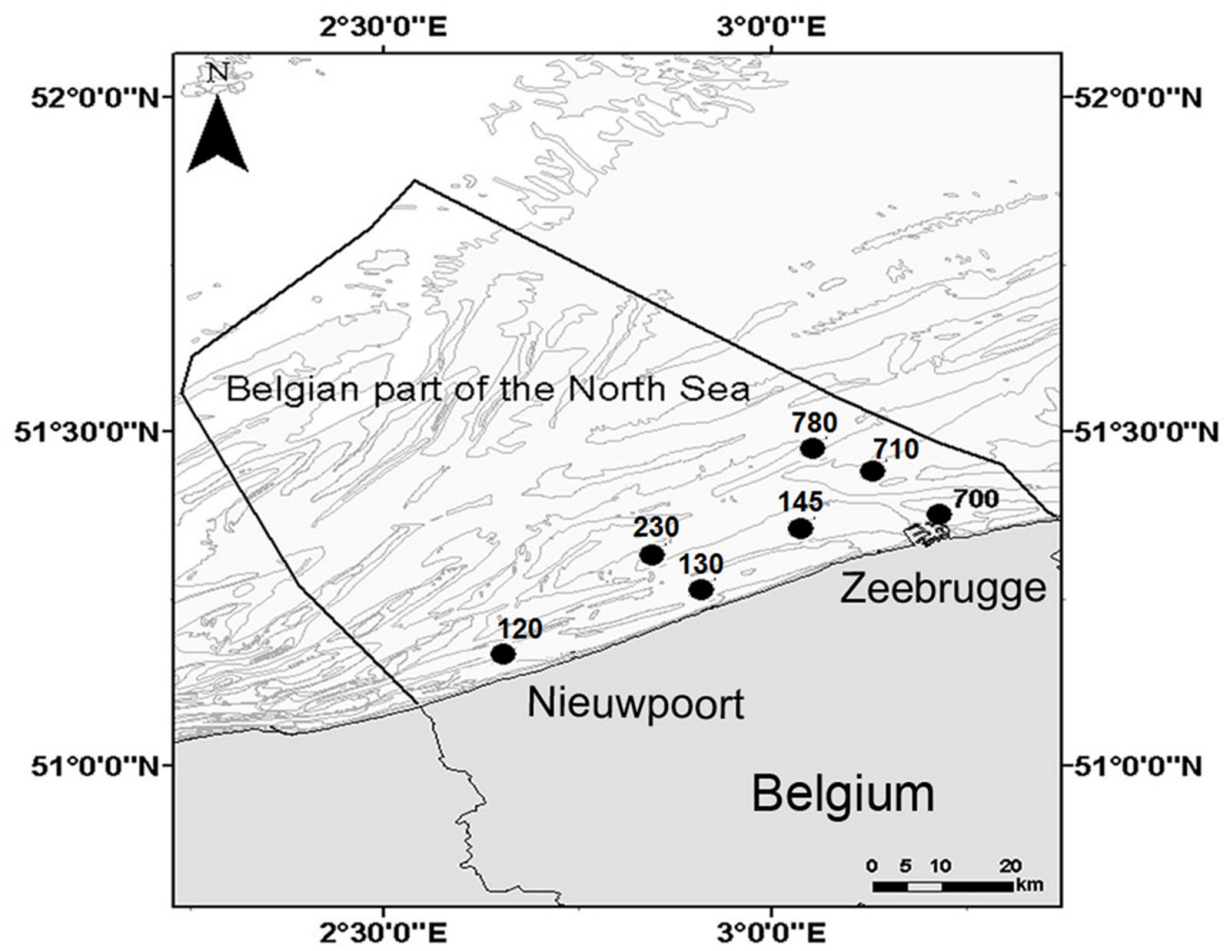
Bathymetry map of the Belgian part of the North Sea with indication of the sampled stations.

In 2011, sediment was collected monthly (February-October) from seven subtidal stations (Fig 1) in the Belgian part of the North Sea (BpNS).

A description of the spatial and temporal patterns (8 months) of environmental variables, macrofaunal community characteristics, N-cycling and details about lab incubations are provided a twin paper [26]. Here, we use a subset of the environmental and macrofaunal data for statistical analysis (S1 Table). In short, sediments in the different stations could be classified as “muddy” (St. 130, 145 and 700), “fine sandy” (St. 120 and 780) and “permeable” sediments (St. 230 and 710). The different sediments were inhabited by different macrofaunal community. Muddy sediments were inhabited by the *Macoma balthica* community (with an average 9 species/0.1 m²), the species rich and abundant *Abra alba* community (with an average 21 species/0.1 m²) was found in the fine sandy sediments, whereas the species poor *Nephtys cirrosa* community (with an average 5 species/0.1 m²) prevailed in the permeable sediments [36]. These differences in communities were reflected in the BPc values: highest values were always found in the fine sandy sediments, the macrofaunal community from the permeable and muddy sediments had comparably low BPc values.

In the water column, chl-*a* concentrations followed clear temporal and spatial patterns previously described in the same area [37,38]. In the nearshore stations (St. 120, 130, 145, 700, 230 and 710), a spring phytoplankton bloom was reflected in strongly elevated chl-*a* concentrations in the water column. In the more offshore stations (St. 780), peak chl-*a* concentrations were observed in late summer. Benthic-pelagic coupling was strong at most stations: highest chl-*a* values in the sediment were always observed shortly after peak values in the water column were observed. This deposition of organic matter triggered mineralization processes in the sediment, which were different according to sediment type. Denitrification rates were highest in the fine sandy sediments throughout the year. During summer, nitrification rates in fine sandy sediments were also higher than in muddy and permeable sediment. In general, nitrification and denitrification rates in permeable and muddy sediments were low throughout the year [26].

Samples for microbial analysis were collected in April (phytoplankton bloom), June (shortly after mass sedimentation of the spring bloom) and September (high mineralization rate; [1]). No specific permits for sampling and ethics requirements were needed since our research was linked to microbiota, and approved by the FWO-research proposal (G.0033.11). The field study did not involve endangered or protected species. Triplicate sediment cores (Plexiglas, internal diameter: 10 cm; height: 25 cm; 3×7 cores per month) were gently inserted in a Reineck Box corer (surface area 180 cm^2^) deployed several times at every station and were half filled with sediment to have an equal proportion of sediment and water inside the cores. The intact sediment cores were transported to a temperature-controlled room on the day of sampling and submerged uncapped in tanks containing continuously aerated seawater at *in situ* salinity. The temperature of the climate room was adjusted to the temperature of seawater recorded by CTD 1 m above the sea floor ranging from 11°C in April to 16°C in June and September. To create water circulation inside the cores, teflon-coated magnets were inserted at appropriate distance from the sediment surface and rotated by a central magnet in the tanks at a speed below the resuspension limit [25]. Every core was aerated separately. Within two days after sampling, the exchanges of dissolved inorganic nitrogen (DIN = NO_3_^-^, NO_2_^-^, NH_4_^+^) and O_2_ across the sediment-water interface were measured during a series of dark incubations (cores were incubated as an average 11h in April, 7h in June and 6h in September to reach steady-state but ensuring oxygen did not drop below 50% saturation) in airtight closed cores as reported in [26]. The sediment-water exchange fluxes of O_2_ and DIN were used to estimate denitrification, nitrification and N-mineralization rates resulting from microbial activities in the sediment using the models described by [25].

At the end of the incubations, cores were sliced. As the annual average maximum oxygen penetration depth (OPD) in all sediment types was less than 1 cm [26], the top 1 cm of sediment cores was homogenized, and subsampled for microbial analyses (using sterilized tools and stored in sterile 50-ml falcon tubes) and environmental variables (using a cut-off syringe to sample 3 to 5 ml of the sediment for the analysis of chl-*a*, phaeophytin, phaeophorbid, % organic carbon [OrgC], % organic nitrogen [OrgN] and grain size). Pigment samples were immediately frozen at -80°C, whereas the samples for grain size and % OrgC and OrgN were dried at 60°C before analysis. Pigments (chl-*a*, phaeophorbid and phaeophytin) were determined by HPLC (Gilson, Middleton, Wisconsin, USA) analysis according to [39]. Total OrgC and OrgN content was analyzed with an Organic Element Analyser (Flash 2000, Thermo Scientific, Wilmington, Delaware, USA) and sediment granulometry by laser diffraction (Malvern Instruments, Malvern, UK). Oxygen samples from the core incubations were analyzed by automated Winkler titration [40], DIN samples were analyzed using automated colorimetric techniques. Oxygen and DIN fluxes were calculated using a mass-balance model [26].

The remaining sediment from the entire core was sieved on a 1mm mesh to retrieve the macrofauna. Macrofauna was sorted, identified to the lowest possible taxonomic level (typically species level), and weighed (as blotted wet weights to determine biomasses) [26]. BPc, calculated taking into account biomass and abundance of each species as well as mobility and sediment reworking traits, was applied as an index to estimate the extent to which a macrofaunal community can affect important ecosystem properties that can affect ecosystem functioning [34,35]. The ratio of phaeopigments to the sum of chl-*a* + phaeopigments (PAP ratio; [41]) and C:N ratio were calculated as an indication of the freshness of the material deposited on the sediment, and was not part of the dataset of [26].

### DNA and RNA extraction, PCR and RT-PCR, DGGE

Extracellular DNA was removed from sediment samples (2.5 g wet weight) as described by [42]. Intracellular DNA was extracted using the Power Soil DNA extraction kit (MO BIO Laboratories, Carlsbad, California, USA). The V3 region of the bacterial 16S rDNA gene was amplified for DGGE using universal bacterial primers (S2 Table). A nested PCR design was used for 16S rDNA amplification of total archaea due to very low yield from direct PCR in some samples [43] (See S1 Text for more details).

To analyze active β-AOB and AOA communities, RNA was extracted from 3.5 g sediment (wet weight) using the RNA Power Soil Total RNA isolation kit (MO BIO Laboratories). RNA samples were reverse transcribed by Omniscript and Sensiscript Reverse Transcriptase Kits (Qiagen, Hilden, Germany) respectively for samples containing ≥ or < 50 ng RNA per reaction. The *amoA* gene was amplified for DGGE using AOA and β-AOB specific primer sets (S2 Table; see also S1 Text).

Denaturating Gradient Gel Electrophoresis (DGGE) is a commonly used technique to characterize microbial community composition and diversity [44–48].

DGGE analysis (S1 Fig) of PCR and RT-PCR amplicons was performed using the DCode Universal Mutation Detection System device (Bio-Rad, Hercules, California, USA). The gels were stained with SYBR gold (Molecular Probes, Invitrogen, Life Technologies) for 30 min followed by visualization and digital capturing of the profiles via the Molecular Imager Gel Doc XR System (Bio-Rad). Digital images were normalized and processed with BioNumerics (version 5.10, Applied Maths). Band analysis was performed by setting background subtraction and least squares filtering according to the instructions of the manufacturer. Each DGGE band was considered to be an operational taxonomic unit (OTU) [49,50]. The relative intensities of the bands in each lane to the total intensity of the lane were used to estimate relative abundance of OTUs [49,50]. Bands were not excised as sequenced as the main focus of the present study was on the effects of macrofauna on microbial taxonomic and functional diversity per se, and not the identity of the OTU’s.

### Data analysis

We used permutational multivariate ANOVA [51] to assess temporal and spatial differences in community composition (based on relative abundance of OTUs), diversity (species richness [S, ‘richness’, number of OTUs] and Shannon-Wiener [H’, log e, ‘diversity’, number and relative abundance of OTUs]) of total bacteria, total archaea and metabolically active β-AOB and AOA. The data set was analyzed using a two-way fixed factor model design. The factors ‘month’ (three levels: April, June, September), ‘station’ (seven levels: 120, 130, 145, 230, 700, 710, 780) and their interactions were tested. Pairwise tests were performed for significant (interaction) terms. Variation in microbial community structure was visualized using Principal Coordinates Analysis (PCO) based on square root transformed data (relative abundance of the band intensity) to remove the contribution of only common species to the similarity [52].

Second-stage MDS, derived from Spearman correlations between pairs of similarity matrices, was applied to visualize interrelationships between multivariate patterns of the different microbial communities, and the macrofaunal community.

We used Distance based Linear Models (DistLM) to investigate the role of measured environmental factors, total macrofaunal density and functional diversity (BPc) in explaining the variation in richness, diversity and community composition of total bacteria and archaea, and active β-AOB and AOA. In a next step, we established the link between abiotic and biotic factors (macrofaunal density and BPc) and attributes of the microbial communities on the one hand and nitrification, denitrification and N-mineralization. All DistLM analyses were performed using the step-wise selection procedures (See S1 Text).

As microbial richness and diversity were highly correlated, they were not incorporated in a single model. Therefore, we ran two different DistLM analyses for each process in the N-cycle. In addition, to test the relationships between organisms and their activities, the nitrification model was run with diversity indices of AOA and β-AOB. As we did not specifically investigate organisms involved in the denitrification process, total communities (bacteria and archaea) were used to construct the best fitted model of denitrification and N-mineralization.

Statistical analyses were performed using Primer v6.1.10 (Primer-E Ltd., Plymouth, United Kingdom) with the PERMANOVA + add-on package [51].

## Results

### Microbial community composition and diversity indices

OTU richness (S) ranged between 13–32 for bacteria, 6–20 for archaea, 0–30 for β-AOB and 0–22 for AOA, while Shannon-Wiener’s index (H’) fluctuated between 2.08–3.24 in bacteria, 1.21–2.67 in archaea, 0–2.97 in β-AOB and 0–2.71 in AOA.

Community composition of total bacteria, total archaea and the β-AOB and AOA were all significantly affected by an interaction between months and stations (term “MoxSt”, S3 Table).

**Fig 2:**
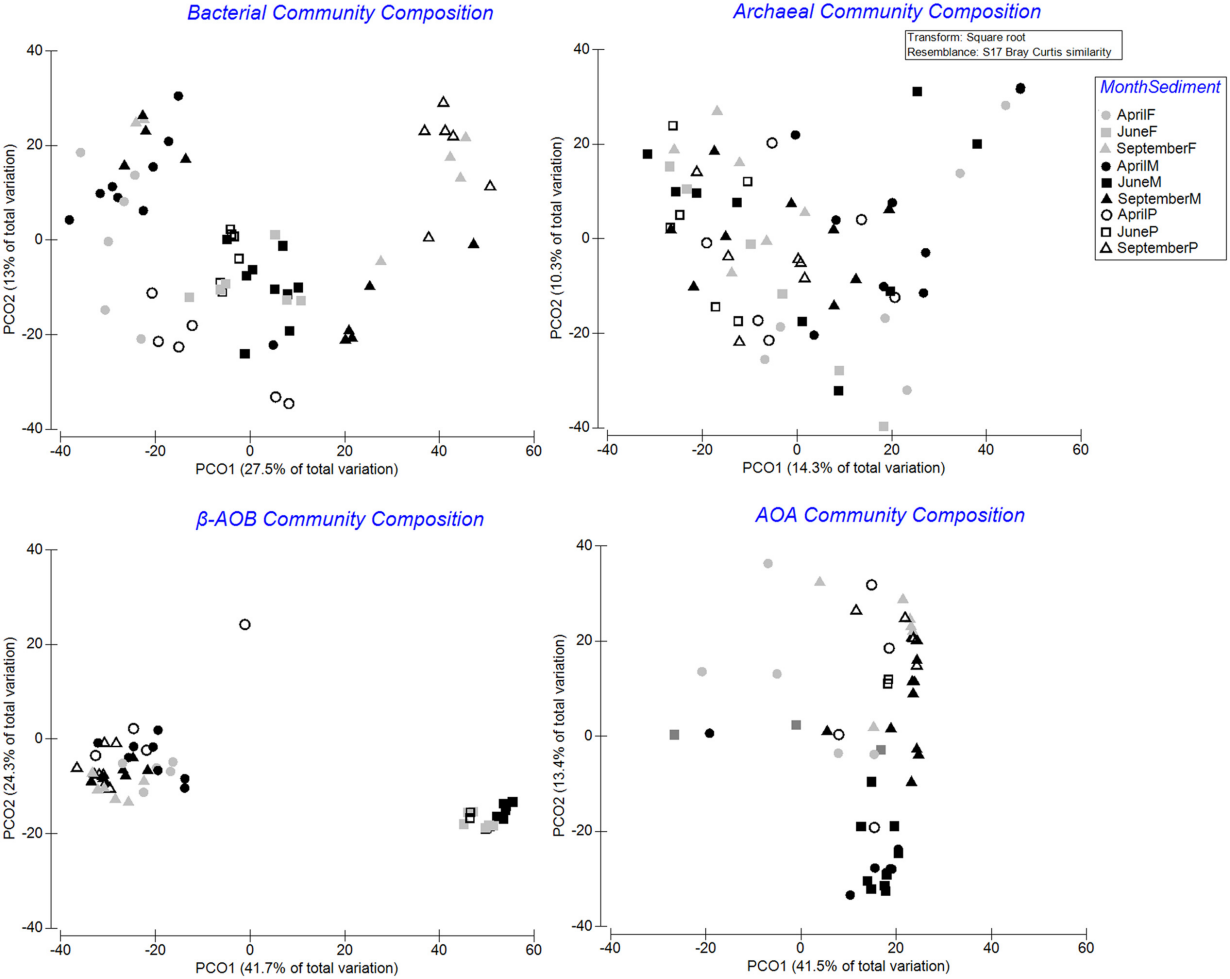
Principal Coordinates Analysis (PCO) of relative abundance data of microbial communities. Data are square root transformed and based on Bray-Curtis similarities. Symbols: April (circle), June (square), September (triangle), muddy stations (black-filled shapes), fine stations (grey-filled shapes), and permeable stations (open shapes).

Pairwise tests (S4 and S5 Tables) and the PCO plots (Fig 2) revealed that the different microbial groups showed different spatial and temporal patterns.

While total archaeal community composition showed limited temporal patterns only in muddy stations, the AOA community in September was separated from June along the second PCO axis (PCO2; 13.4% of the total variation) (pairwise tests, all *P* < 0.05). A seasonal transition was observed in total bacterial and β-AOB community composition (PCO1), which was supported by PERMANOVA (S4 Table). Visualization by PCO also revealed a clear separation in β-AOB community in June, and within-station (muddy St. 130 and 145 and fine sandy St. 780) variation in the community composition of total bacteria in September.

Spatial differences of community composition were present for all investigated groups (total archaea showing limited differences), and mainly in June and September (pairwise tests, all *P* < 0.05). However, consistent pairwise differences were not observed (S5 Table). As stations within each season differed in bacterial and β-AOB community composition mainly along the second axes (PCO2) explaining a lower portion of the total variation than PCO1, it seems community composition in these two groups were more seasonally structured than spatially as evidenced also by a clear separation in June in β-AOB.

Bacterial and AOA richness, and β-AOB and AOA diversity were significantly affected by the interaction term MoxSt. Total archaeal richness and diversity were only affected by “Station”. Richness of β-AOB and diversity of total bacterial community were affected by “Month” and “Station”, however not by their interaction (S3 Table).

**Fig 3:**
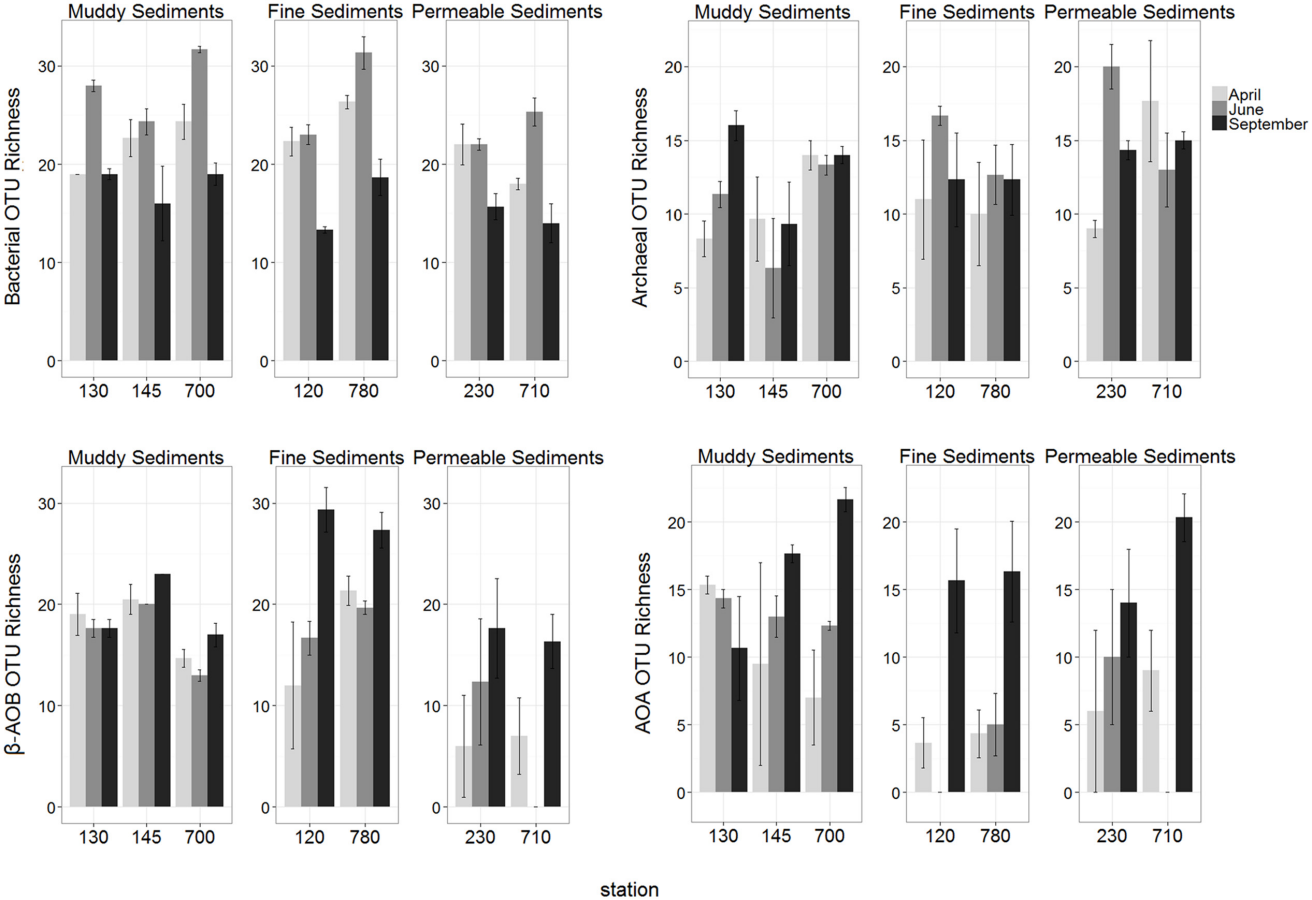
Spatial and temporal variations of OTU richness of all investigated microbial communities (mean ± se).

**Fig 4:**
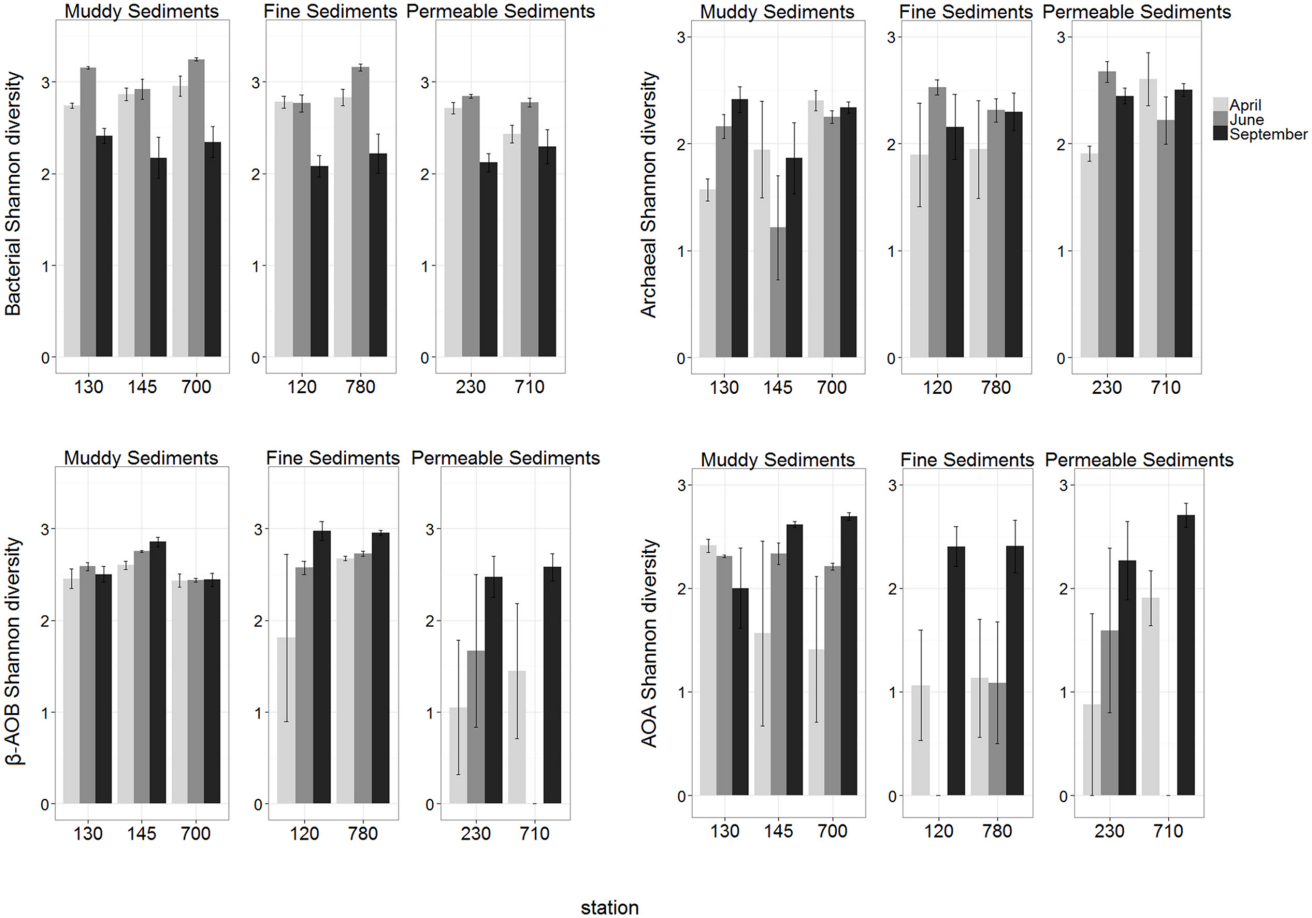
Spatial and temporal variations of Shannon diversity of all investigated microbial communities (mean ± se).

Total bacterial richness and diversity were significantly highest in June in all sediment types while no seasonal difference was observed in total archaea (pairwise tests, all *P* > 0.05; S6 and S7 Tables; Figs 3 and 4).

Investigating spatial differences, generally highest and lowest bacterial richness (mostly present in June) and diversity were observed in muddy and permeable sediments, respectively. Differences between sediment types in archaeal richness and diversity were limited to muddy and permeable sediments and in contrast to bacteria, permeable stations showed generally higher values than muddy stations (pairwise tests, all *P* < 0.05; S8 and S9 Tables; Figs 3 and 4).

In both nitrifying groups (β-AOB and AOA), significantly highest richness values were observed in September in almost all stations. However, timing of highest values for diversity was not consistent for different stations (S6 and S7 Tables; Figs 3 and 4).

Spatial differences in β-AOB richness were lowest in permeable sediments and generally highest in fine sediments. In the latter sediment type, high β-AOB diversity was also obtained in September. Spatial differences per sampling month in AOA richness and diversity were mainly detected in June, when highest values were generally recorded in muddy sediments (pairwise tests, all *P* < 0.05; S8 and S9 Tables; Figs 3 and 4).

**Fig 5:**
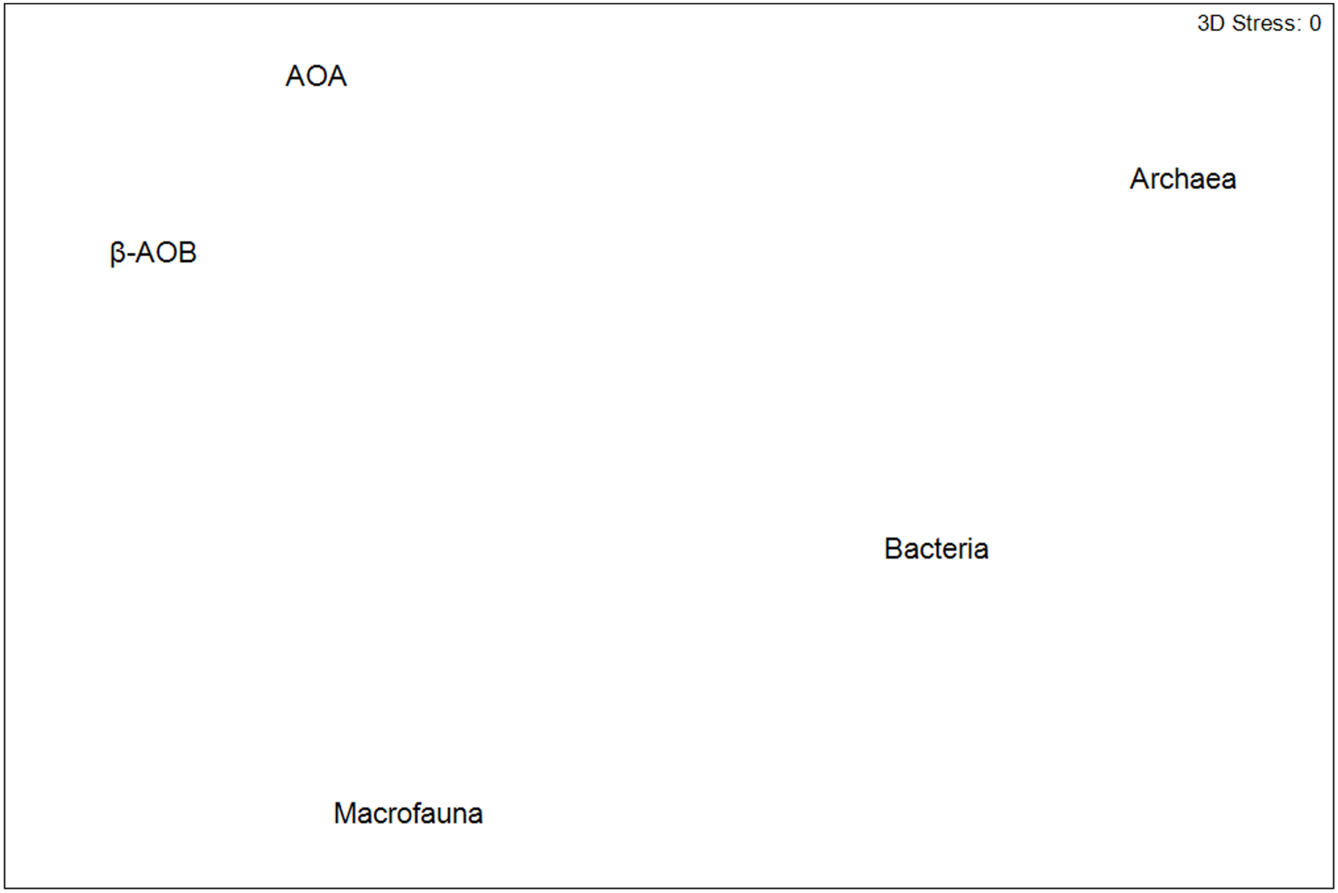
Second stage MDS for community composition of all investigated microbial groups and macrofauna. Data are square root transformed.

Visualization of the similarity of the multivariate patterns of the different microbial groups by second-stage MDS showed that the multivariate patterns for all investigated groups were different but showing more similar patterns in AOA and β-AOB in comparison with those for total bacteria and archaea. The multivariate patterns of the latter groups were different from each other. In addition, the multivariate patterns for all the microbial groups were very different from those observed for the macrofaunal community (Fig 5).

### Linking microbial communities with biotic and abiotic sediment characteristics

Overall, DistLM results (Table 1) indicated that bacterial community composition (total and β-AOB) was better explained by biotic and abiotic sediment characteristics (≈25%) than archaeal community composition (total and AOA; ≈8%). Median grain size (MGS) contributed significantly to the variability of all studied communities. For AOA, this variable was the only significant one in the model while together with MGS, one or more proxies for the quality and quantity of organic matter (chl-*a* concentration, PAP ratio, C:N) significantly contributed to the models of the other groups as well. The macrofaunal bioturbation index, BPc, was only important for the total bacterial community composition.

**Table 1:** Distance-based linear model (DistLM) of microbial community composition and diversity indices against biotic (macrofauna) and abiotic factors.

	*Variable*	*AIC*, *AIC*_*c*_	*SS(trace)*	*Pseudo-F*	*P*	*Prop*	*Cumul*	*res*.*df*
*Bacterial community composition*	*chl-a*	494.31	15850.00	6.40	0.000	0.09	0.09	61
	*MGS* ^*a*^	490.10	14196.00	6.22	0.000	0.08	0.18	60
	*C*:*N ratio*	487.75	9126.90	4.21	0.000	0.05	0.23	59
	*BPc* ^*b*^	487.25	4969.60	2.35	0.012	0.03	0.26	58
*Archaeal community composition*	*chl-a*	507.32	9601.40	3.15	0.000	0.05	0.05	61
	*MGS*	506.79	7307.40	2.46	0.001	0.04	0.09	60
*β-AOB community composition*	*chl-a*	497.12	20553.00	6.99	0.000	0.10	0.10	60
	*C*:*N ratio*	493.93	14175.00	5.15	0.001	0.07	0.18	59
	*MGS*	492.76	8095.70	3.04	0.017	0.04	0.22	58
	*PAP ratio* ^*c*^	491.79	7218.30	2.80	0.029	0.04	0.25	57
*AOA community composition*	*MGS*	494.56	14313.00	5.07	0.001	0.08	0.08	60
*Bacterial OTU richness*	*MGS*	209.85	211.35	7.80	0.007	0.11	0.11	61
	*C*:*N ratio*	203.91	195.91	8.06	0.007	0.10	0.22	60
	*BPc*	198.95	152.50	6.89	0.009	0.08	0.30	59
*Archaeal OTU richness*	*No significant variable*							
*β-AOB OTU richness*	*C*:*N ratio*	250.27	485.45	8.85	0.003	0.13	0.13	60
	*PAP ratio*	247.55	241.53	4.67	0.037	0.06	0.19	59
	*chl-a*	244.23	250.61	5.19	0.028	0.07	0.26	58
*AOA OTU richness*	*chl-a*	244.94	348.07	6.91	0.012	0.10	0.10	60
	*BPc*	241.10	271.39	5.82	0.017	0.08	0.18	59
*Bacterial Shannon diversity*	*MGS*	-123.95	0.94	6.92	0.011	0.10	0.10	61
	*C*:*N ratio*	-129.00	0.87	7.10	0.010	0.09	0.20	60
*Archaeal Shannon diversity*	*No significant variable*							
*β-AOB Shannon diversity*	*C*:*N ratio*	-28.18	10.65	17.32	0.001	0.22	0.22	60
	*chl-a*	-31.64	3.11	5.43	0.025	0.06	0.29	59
	*Macrofauna density*	-34.21	2.40	4.44	0.036	0.05	0.34	58
*AOA Shannon diversity*	*C*:*N ratio*	-1.05	4.77	5.01	0.026	0.08	0.08	60
	*BPc*	-4.22	4.57	5.13	0.026	0.07	0.15	59

Predictor variables subjected to a sequential step-wise selection procedure using the AIC and AIC_c_ criterions for multivariate (community composition) and univariate (richness and diversity) response variables, respectively.

^a^MGS = median grain size.

^b^BPc = Bioturbation Potential of the Community.

^c^PAP ratio = The ratio of phaeopigments to the sum of chl-a + phaeopigments [41].

Evaluating the variables affecting richness and diversity yielded different results (Table 1): macrofaunal densities and/or bioturbation potential (BPc) contributed significantly to diversity aspects (richness and/or diversity) of some microbial groups. Only total archaeal richness and diversity were not affected by macrofauna or any other variables. Generally, the biotic and abiotic variables in the model explained the variation in richness and diversity of total bacteria and β-AOB (20–34%) better than was the case for total archaea and AOA (0–18%). While MGS was incorporated in all models for community composition, it was only part of the model for the total bacterial diversity indices (≈10%).

All other environmental variables in the model were related to quantity and quality of the organic matter. While MGS and C:N were important contributors to the model for total bacterial diversity indices (≈20%), C:N ratio together with chl-*a* concentration were incorporated in the models for β-AOB and AOA richness and/or diversity. PAP ratio contributed significantly only to the β-AOB richness model explaining 6% of the variation.

### Linking the N-cycle with abiotic and biotic factors

DistLM models (Table 2) identified MGS (18% of variation explained), and depending on the model, β-AOB richness or AOA diversity (10 and 6% of the variation explained) as the variables significantly affecting nitrification rates.

**Table 2:** Distance-based linear model (DistLM) of N-cycle processes against biotic (micro- and macrofauna) and abiotic factors.

	*Variable*	*AIC*	*SS(trace)*	*Pseudo-F*	*P*	*Prop*	*Cumul*	*res*.*df*
*Nitrification^1^*	*MGS* ^*a*^	180.55	230.62	12.94	0.000	0.18	0.18	60
	*AOA Shannon diversity*	177.95	76.43	4.54	0.038	0.06	0.24	59
*Nitrification^2^*	*MGS*	180.55	230.62	12.94	0.000	0.18	0.18	60
	*β-AOB OTU richness*	174.70	127.06	7.96	0.007	0.10	0.27	59
*Denitrification^1^*	*BPc* ^*b*^	244.47	1261.60	25.25	0.000	0.30	0.30	60
	*MGS*	241.24	242.26	5.19	0.022	0.06	0.35	59
	*PAP ratio* ^*c*^	237.55	241.86	5.58	0.020	0.06	0.41	58
	*chl-a*	233.81	222.00	5.52	0.019	0.05	0.46	57
*Denitrification^2^*	*BPc*	244.47	1261.60	25.25	0.000	0.30	0.30	60
	*MGS*	241.24	242.26	5.19	0.023	0.06	0.35	59
	*PAP ratio*	237.55	241.86	5.58	0.021	0.06	0.41	58
	*chl-a*	233.81	222.00	5.52	0.018	0.05	0.46	57
*N-mineralization^1^*	*BPc*	70.04	70.95	23.67	0.000	0.28	0.28	60
	*chl-a*	57.75	37.02	15.29	0.000	0.15	0.43	59
	*PAP ratio*	53.13	14.46	6.53	0.013	0.06	0.49	58
	*Bacterial Shannon diversity*	45.97	13.87	7.24	0.009	0.05	0.54	56
*N-mineralization^2^*	*BPc*	70.04	70.95	23.67	0.000	0.28	0.28	60
	*chl-a*	57.75	37.02	15.29	0.000	0.15	0.43	59
	*PAP ratio*	53.13	14.45	6.53	0.015	0.06	0.49	58
	*Bacterial OTU richness*	46.20	13.47	7.01	0.009	0.05	0.54	56

DistLM analyses were run two times for every process separating microbial species richness and diversity in each model (processes run using ^1^Shannon diversity or ^2^OTU richness). Predictor variables subjected to a sequential step-wise selection procedure using the AIC criterion.

^a^MGS = median grain size;

^b^BPc = Bioturbation Potential of the Community;

^c^PAP ratio = The ratio of phaeopigments to the sum of chl-a + phaeopigments [41].

Denitrification could not be explained by the diversity or richness of the total pool of bacteria and archaea; however, bioturbation potential (BPc) significantly affected denitrification rates (30%) together with the abiotic variables MGS, PAP ratio, and chl-*a* concentrations in the sediment, each contributing ≈5% to the models.

Both BPc (28%) and bacterial richness or diversity (≈5%) contributed significantly to the models for total N-mineralization rates. Abiotic variables significantly retained in the model were PAP ratio (6%) and chl-*a* concentration (15%). In total, 54% of the variation in N-mineralization could be explained by these factors.

## Discussion

### Effects of abiotic factors on microbial communities in the sediment

While sediment grain size usually correlates to organic matter content, nutrient concentration and oxygen penetration depth [26,53,54], most studies on microbial communities so far considered granulometric variables in isolation [55]. Sediments with coarser gradients are characterized by low amounts of organic matter especially at top layer and deep oxygen penetration. When sediments become finer, hydrodynamic forces are less strong, hence deposited organic matter can accumulate near the surface [56,57] and result in oxygen stress during periods of intense mineralization [1].

Our study indicated that MGS indeed significantly explained a part of the variation of total bacterial richness and diversity; and community composition of all investigated microbial groups (DistLM; Table 1). Consistent with earlier findings in the BpNS for bacteria [50] and the other studies, generally low richness for bacteria and β-AOB was observed in the surface layer of permeable sediments [50,58,59], which was related to strong hydrodynamic forces (advective currents through the sediment) [50,58,59]. However, this was not the case for AOA and total archaea when richness and diversity of these two groups in permeable sediments were comparable with those in fine sediments in all sampling months (S8 and S9 Tables). Permeable sediments even harboured generally higher values of richness and diversity than muddy sediments in total archaea (Figs 3 and 4). Such differences between archaeal (total or AOA) and bacterial (total or β-AOB) communities were also observed in terms of community composition as permeable sediments showed generally different community composition from the other sediment types in bacterial (total or β-AOB) communities (S5 Table).

Two explanations are possible: *(i)* archaeal communities (total or AOA) are more resistant against hydrodynamic forces by establishing more particle associated rather than free-living communities [60]. *(ii)* These forces do not directly affect microbial communities but do alter concentrations of labile organic matter through washing them out into deeper layers in permeable sediments [56,57]. The latter explanation corroborates earlier studies [61–63] and our findings (DistLM, Table 1) indicating there is a lower dependency on sedimentary abiotic factors (organic matter quantity and quality) in community composition and diversity indices of archaea (total or AOA) compared with bacteria (total or β-AOB). Richness and diversity of total archaea were not even related to any of the measured variables. This suggests that the sedimentation of organic matter and the subsequent changes in the biogeochemical environment have a stronger impact on β-AOB and total bacterial communities in our study area. In addition, this also explains why total bacterial and especially β-AOB community composition varied more seasonally than spatially (visualized by PCO, Fig 2) as proxies of organic matter quantity and quality in the upper cm of sediment show a larger variation temporally than the relatively stable granulometric variables. This was reflected in a clear separation of β-AOB community composition in June (phytoplankton bloom deposition) as chl-*a* and PAP ratio explained higher proportion of the total variation than MGS (14 to 4%) in this group.

The Belgian coastal zone is characterized by high primary production [38,64,65] where 70–75% of the phytoplankton biomass production at the time of spring bloom is under the form (*Phaeocystis colonies*) which is mainly mineralised by bacterial activity [65]. As this reflected seasonal changes in bacterial community composition ([50]; see also Fig 2), the highest bacterial richness and diversity were observed in June (S6 and S7 Tables; Figs 3 and 4). In addition, spatial differences in bacterial richness were also most prominent in June (S8 Table). However, as the obligate chemolithoautotrophic AOB [66] do not directly rely on the availability of organic matter, the sedimentation of the phytoplankton bloom has no direct effect on this community. Therefore, although community composition and diversity indices of total bacteria and β-AOB are both related to organic matter quantity and quality, they showed different patterns (Fig 5). The richness values of metabolically active β-AOB peaked in September (S6 Table; Fig 3). Following the increasing in temperature in the summer time, the degradation of organic matter accelerates in September [1]. This process produces ammonium as a source of energy for nitrifying organisms. The highest richness values of active AOA were also observed in September (second stage MDS showed more similar patterns in AOA and β-AOB in comparison with those for total bacteria and archaea; Fig 5). However, AOA exhibit a variety of metabolic pathways compared to AOB. They are capable to get energy through different carbon-fixing pathways by autotrophic activities [61] as well as the ability of heterotrophic metabolism through oxidizing organic matter [66–68]. Furthermore, there are reports indicating high affinities of AOA to the substrate (ammonium and oxygen) concentration [61,69]. The reasoning above, together with the lower dependency of AOA and total archaea on sedimentary abiotic factors in our study area suggests a general lower spatio-temporal variability in archaeal (total and AOA) than in bacterial (total or β-AOB) communities. Indeed, our results showed that changes in total archaeal community composition and diversity indices were very limited in space and time (no temporal variation in diversity indices). AOA’s did differ seasonally and spatially but were more stable than β-AOB. This is in agreement with findings by [55] in the central part of the North Sea between total bacteria and archaea and earlier studies, which showed that AOA are ubiquitous in sediments, whereas β-AOB did not detect in all the investigated samples [61,70].

### Macrofauna, microbes and the benthic N-cycle

Interactions between macrofaunal and microbial communities are important for biogeochemical processes in coastal benthic ecosystems but have to date been rarely studied [71]. Most research has been based on lab incubations [23,29–31] or focus on the effects of single macrofaunal species in field studies [21,22,24,71]. Results from such small-scale studies have often revealed species-specific differences in these interactions. For example, for some macrofaunal species, microbial communities in burrow walls and surrounding sediments were more similar than communities in burrow walls and surface sediments [21,24,72] while for others burrow wall communities were more similar to those in the surface layer [22,23,73]. As a consequence, the results of these studies cannot readily be translated to complex field situations. In the present study, we adopted an integrated field approach by simultaneously collecting information on macrofauna, important microbial groups and biogeochemical processes over time and in contrasting subtidal sediment types. More specifically, we investigated the effect of the density and bioturbation potential of the whole macrofaunal community on microbial community composition, diversity and N-cycling in these sediments. As the microbial communities were sampled from homogenized sediments from the upper cm layer, small scale distribution patterns will have been disrupted, and specific relationships between macrofauna and certain microbial community descriptors may as a result have gone undetected. Nevertheless, a striking and significant contribution of macrofauna to explaining the overall variation in composition, diversity and richness of different microbial communities was observed. Patterns in macrofaunal community were very different from those for the microbial groups (Fig 5), suggesting that changes in the identity of the macrofaunal species were not reflected in the spatio-temporal patterns of the microbial communities. Richness of β-AOB and diversity of AOA was significantly linked to variation in nitrification rate (DistLM; Table 2). At the same time, a pronounced, significant contribution of macrofaunal abundance and functional diversity was observed explaining respectively the variation in diversity of the metabolically active β-AOB and both richness and diversity of the active AOA (Table 1). While the first finding suggests a biodiversity-ecosystem functioning relationship between ammonia oxidizers and nitrification rates in our study area, the second finding indicates that this relationship is modulated by the activities of the macrofauna.

Intermittent ventilation activities of certain macrofaunal species increase oxygen concentrations during the ventilation and excretion activity, followed by oxygen depletion during resting periods of the animals [28]. As both oxygen and organic matter are required for the nitrification activity of β-AOB and AOA, we suggest that sediment heterogeneity created by the presence of macrofauna lies at the basis of the significant relationship between macrofauna and diversity aspects of the active nitrifying microbes. Indeed, the fauna in our study area does affect sediment oxygenation of the upper cm in fine sandy sediments [25] and redistributes organic matter [25,74]. Strikingly, total archaeal community were not affected by the macrofaunal community (Table 1). The difference observed between the total archaeal community and AOA in our findings revealed that while overviews of total community in relation to macrofauna may be useful, investigation of specific microbial groups are fundamental in establishing a generic mechanistic understanding [31].

The functional index BPc significantly contributed to the models for total bacterial community composition and richness while both BPc and bacterial richness and diversity were significantly linked with N-mineralization (Tables 1 and 2). Macrofauna affect organic matter mineralization by degrading organic matter directly through ingestion [75,76] and through stimulating microbial mineralization [77]. Among the total pool of microbial communities (bacteria and archaea), only aspects of bacterial diversity were significantly linked with N-mineralization rates. Below the oxic layer in marine sediments, organic matter is mineralised mainly by bacteria (i.e. fermenting, denitrifying, sulphate-reducing) [78], of which sulphate-reducing bacteria account for up to 50% of entire organic matter degradation in coastal and shelf ecosystems [78,79]. Annual maximum oxygen penetration depth was only a few mm in muddy and fine sediments, which are characterized by relatively high mineralization rates [26], so that we can expect a more important role of bacteria compared with archaea in mineralization rates.

Assuming that the total bacterial and archaeal communities comprised denitrifiers as well, a significant relationship between these general communities and denitrification could have been detected (Table 2). This was not the case, which can partly be attributed to the fact that we did not target a functional gene for denitrification, and the fact that parts of denitrification occur at deeper sediment layers not included in this study. However, BPc was retained in models for denitrification (Table 2) reflecting an increase in the coupled nitrification-denitrification processes especially in fine sediment as macrofaunal activity increases (characterized by rich functional macrobenthic diversity [26]). Denitrification can be increased by bioturbation and bio-irrigation for two reasons. A first reason is the increase of the surface for coupled nitrification-denitrification [80,81]. A second reason is the increased fluxes of O_2_ and NH_4_ due to bio-irrigation, causing higher nitrification fuelling denitrification [80]. As we did not find a relation between nitrification rates and macrofauna density and bioturbation, we believe that macrofauna activity adds complexity to the biogeochemical settings of the sediment matrix, reflected in an increased surface for the coupled nitrification-denitrification processes.

In conclusion, this study provides evidence to support the link between microbial biodiversity and ecosystem functioning in marine sediments as well as to support the hypothesis that this relationship is modulated by macrofaunal density and functional diversity. Indirect effects of macrofaunal communities on N-cycle processes were found important as well as diversity aspects of microbial communities mediating these processes. As such, our study is major step forward in a general understanding on how marine ecosystem functioning is affected by interactions between organisms with very different body size.

## Supporting Information

S1 Fig Example of DGGE profiles. Molecular markers: lanes M; Bacterial profile (A): lanes 2,3,4 replicates of St. 700 June; lanes 6,7,8, replicates of St. 230 June; lane 9 a replicate of St. 780 September. Archaeal profile (B): lanes 2,3,4 replicates of St. 700 June; lanes 5,6,7 replicates of St.710 June; lanes 9,10,11 replicates of St. 780 June; lanes 12,13,14 replicates of St. 230 June; lane 15 a replicate of St. 145 September. β-AOB and AOA profiles (C and D, respectively): lane 2 a replicate of St.120 September; lanes 3,4,5 replicates of St.780 September; lanes 6,8,9 replicates of St.145 September; lanes 10,11,12 replicates of St. 710 September; lane 13 a replicate of St. 710 June.
(TIF)

S1 Table Spatio-temporal variations in surface sediment (0–1 cm) characteristics, N-cycle processes and macrofaunal characteristics (mean ± se, n = 3); data from [26]. (DOC)

S2 Table Primers used for detection of total archaea, bacteria and ammonia oxidizers (AOA and β-AOB). (DOC)

S3 Table Results of PERMANOVA for microbial communities. Single factor results are only given where the interaction Month x Station (MoxSt) was not significant.
(DOC)

S4 Table Pairwise test results from PERMANOVA analysis for temporal differences of microbial community composition. P-values obtained from Monte-Carlo test, P (MC).
(DOC)

S5 Table Pairwise test results from PERMANOVA analysis for spatial differences of microbial community composition. P-values obtained from Monte-Carlo test, P (MC).
(DOC)

S6 Table Pairwise test results from PERMANOVA analysis for temporal differences of microbial OTU richness. P-values for bacteria and AOA obtained from Monte-Carlo test, P (MC) while those for archaea and β-AOB obtained from permutation, P (Perm).
(DOC)

S7 Table Pairwise test results from PERMANOVA analysis for temporal differences of microbial Shannon diversity. P-values for β-AOB and AOA obtained from Monte-Carlo test, P (MC) while those for bacteria and archaea obtained from permutation, P (Perm).
(DOC)

S8 Table Pairwise test results from PERMANOVA analysis for spatial differences of microbial OTU richness. P-values for bacteria and AOA obtained from Monte-Carlo test, P (MC) while those for archaea and β-AOB obtained from permutation, P (Perm).
(DOC)

S9 Table Pairwise test results from PERMANOVA analysis for spatial differences of microbial Shannon diversity. P-values for β-AOB and AOA obtained from Monte-Carlo test, P (MC) while those for bacteria and archaea obtained from permutation, P (Perm).
(DOC)

S1 Text Supplementary Material and Methods. (DOC)
