# Supplementary figures and images for: The Link between Microbial Diversity and Nitrogen Cycling in Marine Sediments Is Modulated by Macrofaunal Bioturbation
Source: PLoS One. 2015 Jun 23;10(6):e0130116. doi: 10.1371/journal.pone.0130116 (PMC4477903; doi:10.1371/journal.pone.0130116)

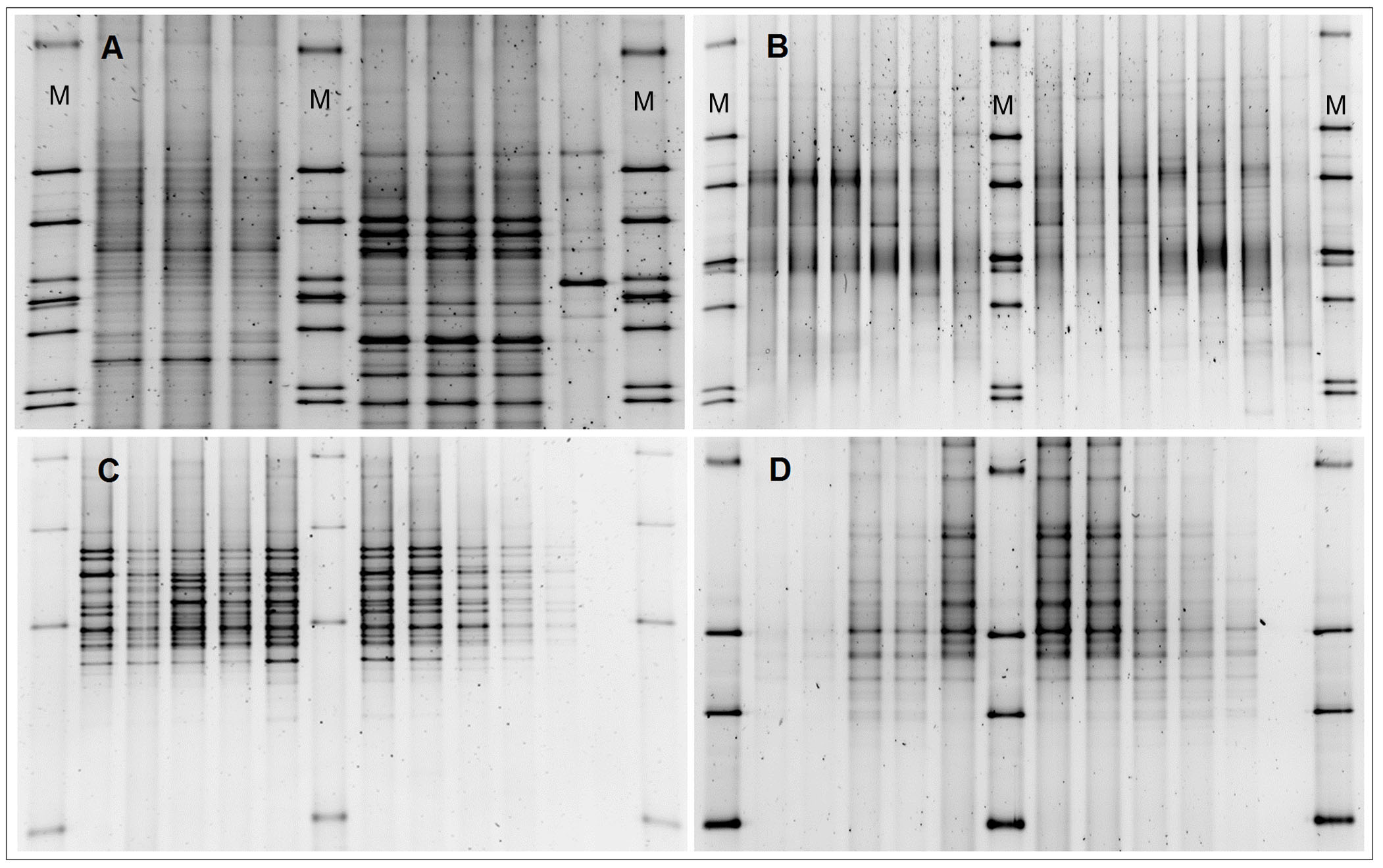

Supplement: S1 Fig — Molecular markers: lanes M; Bacterial profile (A): lanes 2,3,4 replicates of St. 700 June; lanes 6,7,8, replicates of St. 230 June; lane 9 a replicate of St. 780 September. Archaeal profile (B): lanes 2,3,4 replicates of St. 700 June; lanes 5,6,7 replicates of St.710 June; lanes 9,10,11 replicates of St. 780 June; lanes 12,13,14 replicates of St. 230 June; lane 15 a replicate of St. 145 September. β-AOB and AOA profiles (C and D, respectively): lane 2 a replicate of St.120 September; lanes 3,4,5 replicates of St.780 September; lanes 6,8,9 replicates of St.145 September; lanes 10,11,12 replicates of St. 710 September; lane 13 a replicate of St. 710 June. (TIF) [file pone.0130116.s001.tif]
