# Supplementary material for: The Link between Microbial Diversity and Nitrogen Cycling in Marine Sediments Is Modulated by Macrofaunal Bioturbation
Source: PLoS One. 2015 Jun 23;10(6):e0130116. doi: 10.1371/journal.pone.0130116 (PMC4477903; doi:10.1371/journal.pone.0130116)
Supplement: S1 Table — (DOC) [file pone.0130116.s002.doc]

**S1 Table.** **Spatio-temporal variations in surface sediment (0-1 cm) characteristics, N-cycle processes and macrofaunal characteristics (mean ± se, n=3); data from [26].**

|  |  | ***Muddy*** | | | ***Fine Sandy*** | | ***Permeable*** | |
| --- | --- | --- | --- | --- | --- | --- | --- | --- |
| *Stations* |  | **130** | **145** | **700** | **120** | **780** | **230** | **710** |
| *Water depth (m)* |  | 11 | 9 | 12 | 12 | 22 | 14 | 12 |
| **abiotic factors** |  |  |  |  |  |  |  |  |
| *MGS^a^ (μm)* | Apr | 31.44±1.69 | 23.75±2.03 | 26.14±3.93 | 218.01±4.20 | 147.64±1.88 | 232.81±5.85 | 211.86±3.67 |
|  | Jun | 31.56±1.23 | 164.38±2.94 | 26.95±0.33 | 192.25±30.84 | 129.78±4.70 | 259.00±7.71 | 168.39±73.06 |
|  | Sep | 24.11±2.20 | 147.83±28.20 | 29.61±3.50 | 226.60±1.11 | 121.67±5.34 | 242.96±3.63 | 222.21±6.46 |
| *% Silt (<63μm)* | Apr | 70.36±2.09 | 83.16±2.94 | 83.21±4.12 | 12.92±3.14 | 19.84±0.63 | 3.38±3.38 | 0.00±0.00 |
|  | Jun | 68.51±1.94 | 23.85±2.16 | 80.32±0.86 | 20.82±8.55 | 28.02±1.66 | 0.00±0.00 | 27.50±27.50 |
|  | Sep | 80.44±3.90 | 21.16±12.92 | 76.75±3.30 | 12.81±1.69 | 29.03±3.67 | 0.00±0.00 | 0.00±0.00 |
| *% OrgN* | Apr | 0.21±0.01 | 0.26±0.02 | 0.25±0.04 | 0.07±0.01 | 0.05±0.00 | 0.01±0.00 | 0.02±0.00 |
|  | Jun | 0.27±0.04 | 0.07±0.01 | 0.24±0.01 | 0.04±0.00 | 0.08±0.00 | 0.02±0.00 | 0.02±0.00 |
|  | Sep | 0.31±0.01 | 0.06±0.02 | 0.20±0.02 | 0.05±0.00 | 0.09±0.01 | 0.02±0.00 | 0.02±0.00 |
| *% OrgC* | Apr | 1.39±0.01 | 1.84±0.12 | 1.74±0.17 | 0.35±0.09 | 0.37±0.00 | 0.09±0.00 | 0.07±0.00 |
|  | Jun | 2.04±0.30 | 0.48±0.25 | 1.88±0.07 | 0.35±0.02 | 0.23±0.23 | 0.00±0.00 | 0.00±0.00 |
|  | Sep | 2.31±0.04 | 0.48±0.21 | 1.56±0.15 | 0.36±0.04 | 0.44±0.22 | 0.13±0.00 | 0.12±0.01 |
| *Permeability*  *(×10^-12^ m²)* | Apr | 0.00±0.00 | 0.00±0.00 | 0.00±0.00 | 1.20±1.03 | 0.09±0.00 | 5.03±2.23 | 5.74±0.09 |
|  | Jun | 0.00±0.00 | 0.01±0.00 | 0.01±0.00 | 2.57±1.13 | 0.08±0.01 | 7.44±0.11 | 0.03±0.03 |
|  | Sep | 0.00±0.00 | 1.47±1.41 | 0.00±0.00 | 1.01±0.81 | 0.04±0.01 | 7.75±0.22 | 5.79±0.15 |
| *C:N ratio* | Apr | 6.59±0.39 | 7.14±0.43 | 7.21±0.77 | 4.89±0.46 | 6.81±0.69 | 5.19±0.03 | 4.00±0.94 |
|  | Jun | 7.44±0.06 | 6.08±3.11 | 7.75±0.08 | 7.62±0.26 | 3.31±3.31 | 0.00±0.00 | 0.00±0.00 |
|  | Sep | 7.36±0.16 | 7.91±0.54 | 7.70±0.06 | 7.56±0.26 | 4.88±2.46 | 6.68±0.28 | 6.30±0.58 |
| *PAP ratio^b^* | Apr | 0.37±0.27 | 0.07±0.01 | 0.09±0.00 | 0.29±0.04 | 0.13±0.03 | 0.21±0.04 | 0.26±0.02 |
|  | Jun | 0.18±0.02 | 0.28±0.06 | 0.15±0.10 | 0.48±0.05 | 0.32±0.03 | 0.10±0.10 | 0.08±0.08 |
|  | Sep | 0.16±0.01 | 0.19±0.01 | 0.15±0.02 | 0.39±0.04 | 0.40±0.04 | 0.00±0.00 | 0.00±0.00 |
| *Chlorophyll a*  *(µg g^-1^)* | Apr | 0.02±0.01 | 0.02±0.00 | 0.01±0.00 | 0.00±0.00 | 0.00±0.00 | 0.00±0.00 | 0.00±0.00 |
|  | Jun | 25.79±6.09 | 3.53±2.43 | 67.90±50.68 | 0.63±0.07 | 5.12±0.72 | 0.17±0.01 | 0.26±0.09 |
|  | Sep | 18.56±0.71 | 1.84±0.76 | 17.01±3.28 | 0.52±0.10 | 1.91±0.37 | 0.23±0.05 | 0.13±0.01 |
| ***Processes*** |  |  |  |  |  |  |  |  |
| *Nitrification*  *(mmol Nm^-2^d^-1^)* | Apr | -6.16±1.52 | -2.34±0.88 | -5.85±0.89 | -2.34±1.79 | 2.17±0.93 | 0.27±0.31 | 0.92±0.43 |
|  | Jun | -12.80±3.03 | -0.36±0.46 | -3.58±1.58 | -2.48±0.63 | -3.18±0.76 | 0.88±0.37 | -0.22±0.20 |
|  | Sep | 0.10±1.20 | 3.39±0.73 | 2.33±1.05 | 6.32±1.16 | 6.50±0.43 | -0.11±0.23 | 3.40±0.27 |
| *Denitrification*  *(mmol Nm^-2^d^-1^)* | Apr | -5.55±1.28 | 3.21±2.69 | -14.14±10.93 | 8.86±2.27 | 3.16±0.91 | 3.51±1.60 | 2.96±2.32 |
|  | Jun | -10.14±3.15 | 7.53±1.62 | -0.32±1.47 | 17.17±3.84 | 13.48±2.74 | 4.59±0.85 | -0.73±0.65 |
|  | Sep | 4.66±1.78 | 4.44±1.53 | 3.90±1.34 | 10.04±2.16 | 9.49±1.08 | 0.91±0.52 | 3.83±0.35 |
| *N-mineralization*  *(mmol Nm^-2^d^-1^)* | Apr | 1.47±0.18 | 3.16±1.53 | 1.26±0.66 | 1.92±0.16 | 2.29±0.83 | 0.44±0.24 | 0.93±0.43 |
|  | Jun | 2.68±0.81 | 1.56±0.15 | 1.57±0.64 | 4.27±0.97 | 6.31±1.16 | 0.70±0.26 | 0.00±0.00 |
|  | Sep | 4.30±0.31 | 1.93±0.24 | 3.67±0.02 | 5.35±1.57 | 4.52±1.35 | 0.59±0.14 | 1.93±0.25 |
| ***Macrofauna*** |  |  |  |  |  |  |  |  |
| *Biomass*  *(g WW m^-2^)* | Apr | 3.52±3.47 | 0.00±0.00 | 142.45±76.81 | 150.65±77.03 | 283.02±140.49 | 34.72±32.33 | 9.98±9.13 |
|  | Jun | 61.78±33.59 | 73.45±29.36 | 28.64±28.53 | 327.36±157.00 | 392.35±124.03 | 6.40±2.35 | 1.33±1.06 |
|  | Sep | 24.02±12.44 | 81.44±14.87 | 6.14±6.14 | 182.81±70.27 | 328.21±172.24 | 7.74±6.15 | 67.62±66.36 |
| *Density*  *(ind. m^-2^)* | Apr | 84.88±42.44 | 42.44±42.44 | 1443.05±658.89 | 3310.52±1155.34 | 3756.17±1568.51 | 381.98±194.50 | 339.54±84.88 |
|  | Jun | 551.75±185.00 | 551.75±112.29 | 254.65±127.33 | 2758.77±153.03 | 4159.37±1672.04 | 2037.24±530.11 | 339.54±278.31 |
|  | Sep | 127.33±0.00 | 169.77±42.44 | 42.44±42.44 | 2122.13±1181.55 | 5347.76±3014.02 | 1145.95±763.97 | 297.10±42.44 |
| *BPc^c^*  *(m^-2^)* | Apr | 53.47±46.46 | 0.00±0.00 | 909.78±430.05 | 2431.40±580.83 | 2406.82±910.11 | 313.92±213.60 | 317.48±209.26 |
|  | Jun | 531.98±259.66 | 508.61±179.24 | 216.64±202.13 | 4053.77±1553.22 | 4325.42±1445.86 | 466.14±40.81 | 161.04±144.30 |
|  | Sep | 187.21±83.72 | 619.06±176.21 | 64.60±64.60 | 2273.09±494.086 | 3351.80±1687.64 | 430.95±336.15 | 371.66±198.31 |

^a^MGS=median grain size; ^b^PAP ratio=The ratio of phaeopigments to the sum of chl-a + phaeopigments [41]; ^c^BPc= Bioturbation Potential of the Community
