# Supplementary material for: The Link between Microbial Diversity and Nitrogen Cycling in Marine Sediments Is Modulated by Macrofaunal Bioturbation
Source: PLoS One. 2015 Jun 23;10(6):e0130116. doi: 10.1371/journal.pone.0130116 (PMC4477903; doi:10.1371/journal.pone.0130116)
Supplement: S2 Table — (DOC) [file pone.0130116.s003.doc]

**S2 Table. Primers used for detection of total archaea, bacteria and ammonia oxidizers (AOA and β-AOB).**

| *Target gene* | *Primer pair* | *Sequences (5’-3’)* | *Fragment*  *(bp)* | *Reference* |
| --- | --- | --- | --- | --- |
|  |  |  |  |  |
| 16S rDNA Archaea  (1_st_ round) | A8f  A1492r | CGGTTGATCCTGCCGGA  GGCTACCTTGTTACGACTT | 1468 | Casamayor *et al*. (2000)  Casamayor *et al*. (2000) |
| 16S rDNA Archaea  (2_nd_ round) | A344f ^a^  A915r | ACGGGGTGCAGCAGGCGCGA  GTGCTCCCCCGCCAATTCCT | 571 | Perreault *et al*. (2007)  Raskin *et al*. (1994) |
| 16S rDNA Bacteria | F357^a^  R518 | CCTACGGGAGGCAGCAG  ATTACCGCGGCTGCTGG | 161 | Muyzer *et al*. (1993)  Muyzer *et al*. (1993) |
| *amoA* β-AOB | amoA-1F^a^  amoAr-new | GGGGHTTYTACTGGTGGT  CCCCTCBGSAAAVCCTTCTTC | 490 | Hornek *et al*. (2006)  Hornek *et al*. (2006) |
| *amoA* AOA | Arch-amoA-for  Arch-amoA-rev^a^ | CTGAYTGGGCYTGGACATC  TTCTTCTTTGTTGCCCAGTA | 256 | Wuchter *et al*. (2006)  Wuchter *et al*. (2006) |

^a^ To prevent complete melting of amplicons during DGGE, a 40-bp-long GC clamp was attached to the 5' end of one primer. (5'-CGCCCGCCGCGCCCCGCGCCCGGCCCGCCGCCCCCGCCCC-3') attached to Arch-amoA-rev and F357 primers (Muyzer *et al*., 1993); (5'-CGCCCGCCGCGCGCGGCGGGCGGGGCGGGGGCACGGGGGG-3') attached to amoA-1F primer (Hornek *et al*., 2006); (5’-CGCCCGCCGCGCCCCGCGCCCGGCCCGCCGCCCCCGCCCG-3’) attached to A344f primer (Perreault *et al*., 2007).
