# Supplementary material for: The Link between Microbial Diversity and Nitrogen Cycling in Marine Sediments Is Modulated by Macrofaunal Bioturbation
Source: PLoS One. 2015 Jun 23;10(6):e0130116. doi: 10.1371/journal.pone.0130116 (PMC4477903; doi:10.1371/journal.pone.0130116)
Supplement: S3 Table — Single factor results are only given where the interaction Month x Station (MoxSt) was not significant. (DOC) [file pone.0130116.s004.doc]

**S3 Table. Results of PERMANOVA for microbial communities.**

|  | *factor* | *df_term_* | *df_error_* | *Pseudo-F* | *P(perm)* |
| --- | --- | --- | --- | --- | --- |
| *Bacterial community composition* | MoxSt | 12 | 42 | 3.21 | 0.000 |
| *Archaeal community composition* | MoxSt | 12 | 42 | 2.06 | 0.000 |
| *β-AOB community composition* | MoxSt | 12 | 42 | 3.03 | 0.000 |
| *AOA community composition* | MoxSt | 12 | 42 | 2.79 | 0.000 |
|  |  |  |  |  |  |
| *Bacterial OTU richness* | MoxSt | 12 | 42 | 2.01 | 0.048 |
| *Archaeal OTU richness* | St | 6 | 42 | 2.98 | 0.023 |
| *β-AOB OTU richness* | St  Mo | 6  2 | 42 | 10.05  13.25 | 0.001  0.001 |
| *AOA OTU richness* | MoxSt | 12 | 42 | 2.46 | 0.018 |
|  |  |  |  |  |  |
| *Bacterial Shannon diversity* | St  Mo | 6  2 | 42 | 4.16  83.63 | 0.002  0.001 |
| *Archaeal Shannon diversity* | St | 6 | 42 | 3.11 | 0.022 |
| *β-AOB Shannon diversity* | MoxSt | 12 | 42 | 2.18 | 0.038 |
| *AOA Shannon diversity* | MoxSt | 12 | 42 | 2.56 | 0.024 |

Single factor results are only given where the interaction Month x Station (MoxSt) was not significant.
