# Supplementary material for: The Link between Microbial Diversity and Nitrogen Cycling in Marine Sediments Is Modulated by Macrofaunal Bioturbation
Source: PLoS One. 2015 Jun 23;10(6):e0130116. doi: 10.1371/journal.pone.0130116 (PMC4477903; doi:10.1371/journal.pone.0130116)
Supplement: S4 Table — P-values obtained from Monte-Carlo test, P (MC). (DOC) [file pone.0130116.s005.doc]

**S4 Table. Pairwise test results from PERMANOVA analysis for temporal differences of microbial community composition.**

| ***Community composition* Muddy stations** | | | | | | **Fine sandy stations** | | | | | **Permeable stations** | | | |
| --- | --- | --- | --- | --- | --- | --- | --- | --- | --- | --- | --- | --- | --- | --- |
|  | 130 | | 145 | | 700 | | 120 | | 780 | | 230 | | 710 | |
| **Bacteria** | t | P | t | P | t | P | t | P | t | P | t | P | t | P |
| April-June | 2.55 | **0.017** | 1.97 | **0.041** | 1.40 | 0.110 | 2.23 | **0.024** | 2.07 | **0.033** | 3.21 | **0.012** | 3.70 | **0.004** |
| April-Sept | 1.97 | **0.046** | 1.38 | 0.173 | 1.64 | 0.088 | 3.19 | **0.005** | 1.45 | 0.145 | 4.14 | **0.003** | 3.32 | **0.005** |
| June-Sept | 2.23 | **0.032** | 1.60 | 0.100 | 2.25 | **0.023** | 3.51 | **0.005** | 1.76 | 0.083 | 9.31 | **0.000** | 3.75 | **0.006** |
|  |  |  |  |  |  |  |  |  |  |  |  |  |  |  |
| **Archaea** |  |  |  |  |  |  |  |  |  |  |  |  |  |  |
|  |  |  |  |  |  |  |  |  |  |  |  |  |  |  |
| April-June | 1.92 | **0.046** | 1.58 | 0.094 | 2.87 | **0.010** | 1.20 | 0.256 | 1.59 | 0.103 | 1.34 | 0.171 | 1.45 | 0.130 |
| April-Sept | 1.10 | 0.054 | 1.10 | 0.348 | 2.37 | **0.018** | 1.28 | 0.207 | 1.36 | 0.163 | 0.97 | 0.46 | 1.81 | 0.050 |
| June-Sept | 2.20 | **0.029** | 1.01 | 0.344 | 1.63 | 0.093 | 1.18 | 0.281 | 1.79 | 0.062 | 0.84 | 0.604 | 1.60 | 0.086 |
|  |  |  |  |  |  |  |  |  |  |  |  |  |  |  |
| **β-AOB** |  |  |  |  |  |  |  |  |  |  |  |  |  |  |
|  |  |  |  |  |  |  |  |  |  |  |  |  |  |  |
| April-June | 3.83 | **0.004** | 4.95 | **0.004** | 5.03 | **0.001** | 2.31 | **0.029** | 6.46 | **0.000** | 1.13 | 0.317 | 1.91 | 0.113 |
| April-Sept | 2.27 | **0.025** | 2.77 | **0.022** | 3.17 | **0.011** | 1.14 | 0.318 | 2.14 | **0.034** | 1.44 | 0.172 | 1.22 | 0.258 |
| June-Sept | 7.16 | **0.000** | 8.97 | **0.000** | 12.11 | **0.000** | 4.00 | **0.003** | 5.84 | **0.000** | 2.28 | **0.028** | 4.33 | **0.002** |
|  |  |  |  |  |  |  |  |  |  |  |  |  |  |  |
| **AOA** |  |  |  |  |  |  |  |  |  |  |  |  |  |  |
|  |  |  |  |  |  |  |  |  |  |  |  |  |  |  |
| April-June | 4.41 | **0.003** | 1.62 | 0.149 | 1.54 | 0.131 | 1.83 | 0.122 | 1.17 | 0.281 | 0.88 | 0.442 | 2.93 | **0.009** |
| April-Sept | 2.10 | **0.036** | 1.72 | 0.138 | 1.55 | 0.138 | 1.75 | 0.071 | 1.29 | 0.233 | 1.79 | 0.116 | 1.30 | 0.227 |
| June-Sept | 2.86 | **0.009** | 4.83 | **0.003** | 3.73 | **0.004** | 5.96 | **0.001** | 2.07 | **0.043** | 1.28 | 0.239 | 10.74 | **0.000** |
|  |  |  |  |  |  |  |  |  |  |  |  |  |  |  |

P-values obtained from Monte-Carlo test, P (MC).
