# Supplementary material for: The Link between Microbial Diversity and Nitrogen Cycling in Marine Sediments Is Modulated by Macrofaunal Bioturbation
Source: PLoS One. 2015 Jun 23;10(6):e0130116. doi: 10.1371/journal.pone.0130116 (PMC4477903; doi:10.1371/journal.pone.0130116)
Supplement: S5 Table — P-values obtained from Monte-Carlo test, P (MC). (DOC) [file pone.0130116.s006.doc]

**S5 Table. Pairwise test results from PERMANOVA analysis for spatial differences of microbial community composition.**

| ***Community composition*** **Muddy stations** | | | | | | | | **Fine sandy stations** | | | | **Permeable stations** | | | |
| --- | --- | --- | --- | --- | --- | --- | --- | --- | --- | --- | --- | --- | --- | --- | --- |
|  | | 130 | | 145 | | 700 | | 120 | | 780 | | 230 | | 710 | |
| **Bacteria** | | t | P | t | P | t | P | t | P | t | P | t | P | t | P |
|  | 130 |  |  |  |  |  |  |  |  |  |  |  |  |  |  |
|  | 145 | 1.47 | 0.148 |  |  |  |  |  |  |  |  |  |  |  |  |
| April | 700 | 1.04 | 0.385 | 0.86 | 0.534 |  |  |  |  |  |  |  |  |  |  |
|  | 120 | 1.81 | 0.068 | 1.04 | 0.391 | 1.10 | 0.345 |  |  |  |  |  |  |  |  |
|  | 780 | 1.61 | 0.106 | 1.72 | 0.064 | 1.14 | 0.309 | 1.37 | 0.184 |  |  |  |  |  |  |
|  | 230 | 2.45 | **0.017** | 1.92 | **0.047** | 1.55 | 0.118 | 1.55 | 0.124 | 2.02 | **0.034** |  |  |  |  |
|  | 710 | 2.94 | **0.009** | 2.66 | **0.014** | 1.93 | 0.057 | 2.28 | **0.028** | 2.22 | **0.029** | 1.88 | 0.065 |  |  |
|  |  |  |  |  |  |  |  |  |  |  |  |  |  |  |  |
|  | 130 |  |  |  |  |  |  |  |  |  |  |  |  |  |  |
|  | 145 | 1.71 | 0.103 |  |  |  |  |  |  |  |  |  |  |  |  |
| June | 700 | 2.26 | **0.030** | 1.90 | 0.068 |  |  |  |  |  |  |  |  |  |  |
|  | 120 | 2.80 | **0.010** | 1.26 | 0.233 | 2.97 | **0.012** |  |  |  |  |  |  |  |  |
|  | 780 | 3.07 | **0.008** | 2.08 | **0.035** | 3.22 | **0.007** | 2.82 | **0.010** |  |  |  |  |  |  |
|  | 230 | 5.56 | **0.002** | 2.37 | **0.025** | 5.91 | **0.001** | 2.76 | **0.012** | 3.07 | **0.009** |  |  |  |  |
|  | 710 | 4.62 | **0.003** | 2.26 | **0.027** | 4.91 | **0.002** | 2.85 | **0.011** | 2.70 | **0.013** | 2.86 | **0.011** |  |  |
|  |  |  |  |  |  |  |  |  |  |  |  |  |  |  |  |
|  | 130 |  |  |  |  |  |  |  |  |  |  |  |  |  |  |
|  | 145 | 0.92 | 0.452 |  |  |  |  |  |  |  |  |  |  |  |  |
| Sept | 700 | 1.42 | 0.166 | 1.33 | 0.207 |  |  |  |  |  |  |  |  |  |  |
|  | 120 | 2.73 | **0.017** | 1.75 | 0.114 | 2.44 | **0.022** |  |  |  |  |  |  |  |  |
|  | 780 | 0.69 | 0.609 | 0.67 | 0.655 | 1.50 | 0.134 | 2.01 | 0.055 |  |  |  |  |  |  |
|  | 230 | 2.88 | **0.012** | 1.74 | 0.127 | 2.62 | **0.021** | 1.25 | 0.237 | 2.05 | 0.055 |  |  |  |  |
|  | 710 | 2.43 | **0.024** | 1.57 | 0.143 | 2.13 | **0.038** | 2.33 | **0.021** | 1.86 | 0.069 | 1.62 | 0.123 |  |  |
|  |  |  |  |  |  |  |  |  |  |  |  |  |  |  |  |
|  |  |  |  |  |  |  |  |  |  |  |  |  |  |  |  |
|  |  |  |  |  |  |  |  |  |  |  |  |  |  |  |  |
| **Archaea** | |  |  |  |  |  |  |  |  |  |  |  |  |  |  |
|  | 130 |  |  |  |  |  |  |  |  |  |  |  |  |  |  |
|  | 145 | 1.50 | 0.131 |  |  |  |  |  |  |  |  |  |  |  |  |
| April | 700 | 1.89 | 0.060 | 1.57 | 0.117 |  |  |  |  |  |  |  |  |  |  |
|  | 120 | 1.06 | 0.374 | 1.25 | 0.225 | 1.70 | 0.070 |  |  |  |  |  |  |  |  |
|  | 780 | 1.56 | 0.118 | 1.27 | 0.240 | 1.37 | 0.170 | 1.25 | 0.229 |  |  |  |  |  |  |
|  | 230 | 1.33 | 0.206 | 1.32 | 0.201 | 1.51 | 0.123 | 1.21 | 0.255 | 0.94 | 0.500 |  |  |  |  |
|  | 710 | 1.82 | 0.061 | 1.54 | 0.112 | 1.78 | 0.060 | 1.49 | 0.115 | 1.19 | 0.271 | 1.31 | 0.191 |  |  |
|  |  |  |  |  |  |  |  |  |  |  |  |  |  |  |  |
|  | 130 |  |  |  |  |  |  |  |  |  |  |  |  |  |  |
|  | 145 | 1.55 | 0.112 |  |  |  |  |  |  |  |  |  |  |  |  |
| June | 700 | 2.37 | **0.015** | 1.91 | 0.073 |  |  |  |  |  |  |  |  |  |  |
|  | 120 | 1.03 | 0.391 | 1.47 | 0.147 | 1.97 | **0.041** |  |  |  |  |  |  |  |  |
|  | 780 | 2.43 | **0.018** | 2.20 | **0.031** | 2.03 | **0.041** | 1.86 | **0.047** |  |  |  |  |  |  |
|  | 230 | 1.84 | **0.044** | 1.99 | **0.043** | 1.76 | 0.066 | 1.21 | 0.254 | 1.51 | 0.122 |  |  |  |  |
|  | 710 | 1.82 | **0.046** | 1.38 | 0.172 | 1.36 | 0.198 | 1.41 | 0.159 | 1.25 | 0.232 | 1.08 | 0.359 |  |  |
|  |  |  |  |  |  |  |  |  |  |  |  |  |  |  |  |
|  | 130 |  |  |  |  |  |  |  |  |  |  |  |  |  |  |
|  | 145 | 1.25 | 0.248 |  |  |  |  |  |  |  |  |  |  |  |  |
| Sept | 700 | 2.32 | **0.018** | 1.00 | 0.427 |  |  |  |  |  |  |  |  |  |  |
|  | 120 | 1.53 | 0.113 | 0.70 | 0.741 | 0.95 | 0.477 |  |  |  |  |  |  |  |  |
|  | 780 | 1.73 | 0.062 | 0.99 | 0.438 | 1.40 | 0.146 | 0.96 | 0.470 |  |  |  |  |  |  |
|  | 230 | 1.67 | 0.081 | 0.77 | 0.684 | 0.87 | 0.544 | 0.61 | 0.830 | 1.06 | 0.367 |  |  |  |  |
|  | 710 | 2.10 | **0.030** | 1.34 | 0.176 | 1.78 | 0.061 | 1.55 | 0.103 | 1.53 | 0.105 | 1.40 | 0.147 |  |  |

P-values obtained from Monte-Carlo test, P (MC).

**Table S4 (continued):**

| ***Community composition*** **Muddy stations** | | | | | | | | **Fine sandy stations** | | | | **Permeable stations** | | | |
| --- | --- | --- | --- | --- | --- | --- | --- | --- | --- | --- | --- | --- | --- | --- | --- |
|  | | 130 | | 145 | | 700 | | 120 | | 780 | | 230 | | 710 | |
| **β-AOB** | | t | P | t | P | t | P | t | P | t | P | t | P | t | P |
|  | 130 |  |  |  |  |  |  |  |  |  |  |  |  |  |  |
|  | 145 | 1.68 | 0.113 |  |  |  |  |  |  |  |  |  |  |  |  |
| April | 700 | 0.59 | 0.758 | 2.15 | **0.048** |  |  |  |  |  |  |  |  |  |  |
|  | 120 | 1.40 | 0.176 | 1.12 | 0.336 | 1.62 | 0.108 |  |  |  |  |  |  |  |  |
|  | 780 | 1.71 | 0.091 | 2.69 | **0.027** | 1.89 | 0.064 | 1.51 | 0.147 |  |  |  |  |  |  |
|  | 230 | 1.05 | 0.381 | 1.22 | 0.280 | 1.21 | 0.261 | 0.73 | 0.622 | 1.54 | 0.115 |  |  |  |  |
|  | 710 | 1.70 | 0.074 | 1.26 | 0.255 | 1.91 | 0.054 | 0.48 | 0.789 | 1.77 | 0.074 | 0.81 | 0.568 |  |  |
|  |  |  |  |  |  |  |  |  |  |  |  |  |  |  |  |
|  | 130 |  |  |  |  |  |  |  |  |  |  |  |  |  |  |
|  | 145 | 1.50 | 0.152 |  |  |  |  |  |  |  |  |  |  |  |  |
| June | 700 | 2.54 | **0.022** | 5.64 | **0.001** |  |  |  |  |  |  |  |  |  |  |
|  | 120 | 4.42 | **0.002** | 5.12 | **0.002** | 7.44 | **0.001** |  |  |  |  |  |  |  |  |
|  | 780 | 1.67 | 0.095 | 1.07 | 0.368 | 4.94 | **0.001** | 4.48 | **0.004** |  |  |  |  |  |  |
|  | 230 | 1.30 | 0.241 | 1.25 | 0.261 | 1.36 | 0.222 | 1.29 | 0.241 | 1.21 | 0.276 |  |  |  |  |
|  | 710 | 13.4 | **0.000** | 33.89 | **0.000** | 39.49 | **0.000** | 16.90 | **0.000** | 24.26 | **0.000** | 1.96 | 0.112 |  |  |
|  |  |  |  |  |  |  |  |  |  |  |  |  |  |  |  |
|  | 130 |  |  |  |  |  |  |  |  |  |  |  |  |  |  |
|  | 145 | 2.94 | **0.010** |  |  |  |  |  |  |  |  |  |  |  |  |
| Sept | 700 | 1.58 | 0.103 | 4.64 | **0.001** |  |  |  |  |  |  |  |  |  |  |
|  | 120 | 1.95 | 0.054 | 1.25 | 0.250 | 2.44 | **0.022** |  |  |  |  |  |  |  |  |
|  | 780 | 2.23 | **0.033** | 2.11 | **0.043** | 2.51 | **0.021** | 1.06 | 0.358 |  |  |  |  |  |  |
|  | 230 | 2.50 | **0.022** | 1.64 | 0.105 | 3.47 | **0.007** | 1.46 | 0.155 | 2.18 | **0.030** |  |  |  |  |
|  | 710 | 1.81 | 0.070 | 1.09 | 0.340 | 2.22 | **0.032** | 0.74 | 0.630 | 1.15 | 0.303 | 1.06 | 0.364 |  |  |
|  |  |  |  |  |  |  |  |  |  |  |  |  |  |  |  |
|  |  |  |  |  |  |  |  |  |  |  |  |  |  |  |  |
|  |  |  |  |  |  |  |  |  |  |  |  |  |  |  |  |
| **AOA** | |  |  |  |  |  |  |  |  |  |  |  |  |  |  |
|  | 130 |  |  |  |  |  |  |  |  |  |  |  |  |  |  |
|  | 145 | 1.31 | 0.270 |  |  |  |  |  |  |  |  |  |  |  |  |
| April | 700 | 1.41 | 0.191 | 0.85 | 0.547 |  |  |  |  |  |  |  |  |  |  |
|  | 120 | 2.31 | **0.026** | 1.13 | 0.343 | 1.22 | 0.265 |  |  |  |  |  |  |  |  |
|  | 780 | 1.65 | 0.091 | 0.97 | 0.453 | 1.05 | 0.376 | 1.14 | 0.313 |  |  |  |  |  |  |
|  | 230 | 2.26 | **0.043** | 1.15 | 0.317 | 0.99 | 0.404 | 0.96 | 0.423 | 1.32 | 0.197 |  |  |  |  |
|  | 710 | 1.66 | 0.096 | 0.96 | 0.462 | 1.12 | 0.321 | 1.40 | 0.164 | 0.79 | 0.631 | 1.37 | 0.201 |  |  |
|  |  |  |  |  |  |  |  |  |  |  |  |  |  |  |  |
|  | 130 |  |  |  |  |  |  |  |  |  |  |  |  |  |  |
|  | 145 | 3.56 | **0.013** |  |  |  |  |  |  |  |  |  |  |  |  |
| June | 700 | 1.87 | 0.072 | 2.60 | **0.021** |  |  |  |  |  |  |  |  |  |  |
|  | 120 | 15.43 | **0.000** | 8.06 | **0.000** | 6.27 | **0.000** |  |  |  |  |  |  |  |  |
|  | 780 | 2.12 | **0.045** | 1.34 | 0.217 | 1.99 | **0.046** | 2.65 | **0.016** |  |  |  |  |  |  |
|  | 230 | 1.93 | 0.071 | 1.10 | 0.346 | 1.67 | 0.099 | 2.00 | 0.115 | 0.88 | 0.509 |  |  |  |  |
|  | 710 | 15.43 | **0.000** | 8.06 | **0.000** | 6.27 | **0.001** | 2.00 | 0.110 | 2.65 | **0.015** | 2.00 | 0.120 |  |  |
|  |  |  |  |  |  |  |  |  |  |  |  |  |  |  |  |
|  | 130 |  |  |  |  |  |  |  |  |  |  |  |  |  |  |
|  | 145 | 1.82 | 0.091 |  |  |  |  |  |  |  |  |  |  |  |  |
| Sept | 700 | 1.67 | 0.097 | 5.75 | **0.001** |  |  |  |  |  |  |  |  |  |  |
|  | 120 | 1.90 | 0.062 | 3.22 | **0.012** | 3.22 | **0.009** |  |  |  |  |  |  |  |  |
|  | 780 | 1.48 | 0.148 | 1.61 | 0.141 | 3.10 | **0.010** | 1.66 | 0.109 |  |  |  |  |  |  |
|  | 230 | 1.21 | 0.274 | 2.33 | **0.031** | 2.55 | **0.022** | 1.60 | 0.123 | 1.50 | 0.173 |  |  |  |  |
|  | 710 | 2.05 | **0.047** | 3.02 | **0.013** | 4.07 | **0.004** | 1.71 | 0.112 | 0.99 | 0.391 | 1.69 | 0.099 |  |  |

P-values obtained from Monte-Carlo test, P (MC).
