# Supplementary material for: The Link between Microbial Diversity and Nitrogen Cycling in Marine Sediments Is Modulated by Macrofaunal Bioturbation
Source: PLoS One. 2015 Jun 23;10(6):e0130116. doi: 10.1371/journal.pone.0130116 (PMC4477903; doi:10.1371/journal.pone.0130116)
Supplement: S6 Table — P-values for bacteria and AOA obtained from Monte-Carlo test, P (MC) while those for archaea and β-AOB obtained from permutation, P (Perm). (DOC) [file pone.0130116.s007.doc]

***S6 Table. Pairwise test results from PERMANOVA analysis for temporal differences of microbial OTU richness.***

| ***OTU richness* Muddy stations** | | | | | | **Fine sandy stations** | | | | | **Permeable stations** | | | |
| --- | --- | --- | --- | --- | --- | --- | --- | --- | --- | --- | --- | --- | --- | --- |
|  | 130 | | 145 | | 700 | | 120 | | 780 | | 230 | | 710 | |
| **Bacteria** | t | P | t | P | t | P | t | P | t | P | t | P | t | P |
| April-June | 15.59 | **0.000** | 0.73 | 0.515 | 4.08 | **0.014** | 0.38 | 0.724 | 2.78 | 0.052 | 2.52 | 0.065 | 4.69 | **0.009** |
| April-Sept | 10.25 | **0.000** | 1.58 | 0.190 | 2.53 | 0.067 | 6.04 | **0.005** | 3.89 | **0.018** | 2.56 | 0.058 | 1.92 | 0.126 |
| June-Sept | 11.02 | **0.000** | 2.07 | 0.103 | 10.54 | **0.000** | 9.17 | **0.001** | 5.08 | **0.007** | 4.36 | **0.011** | 4.58 | **0.009** |
|  |  |  |  |  |  |  |  |  |  |  |  |  |  |  |
|  |  |  |  |  |  |  |  |  |  |  |  |  |  |  |
| **AOA** |  |  |  |  |  |  |  |  |  |  |  |  |  |  |
|  |  |  |  |  |  |  |  |  |  |  |  |  |  |  |
| April-June | 1.06 | 0.330 | 0.60 | 0.602 | 1.51 | 0.187 | 1.98 | 0.126 | 0.23 | 0.835 | 0.51 | 0.618 | 3.00 | **0.030** |
| April-Sept | 1.12 | 0.302 | 1.44 | 0.267 | 4.05 | **0.011** | 2.81 | **0.047** | 2.92 | **0.038** | 1.11 | 0.328 | 3.26 | **0.033** |
| June-Sept | 0.94 | 0.396 | 2.80 | **0.044** | 9.90 | **0.002** | 4.07 | **0.014** | 2.59 | 0.054 | 0.62 | 0.601 | 11.53 | **0.002** |
|  |  |  |  |  |  |  |  |  |  |  |  |  |  |  |
| **Archaea** | t | P |  |  |  |  |  |  |  |  |  |  |  |  |
|  |  |  |  |  |  |  |  |  |  |  |  |  |  |  |
| April-June | 1.51 | 0.136 |  |  |  |  |  |  |  |  |  |  |  |  |
| April-Sept | 1.50 | 0.136 |  |  |  |  |  |  |  |  |  |  |  |  |
| June-Sept | 0.00 | 1.000 |  |  |  |  |  |  |  |  |  |  |  |  |
|  |  |  |  |  |  |  |  |  |  |  |  |  |  |  |
| **β-AOB** |  |  |  |  |  |  |  |  |  |  |  |  |  |  |
|  |  |  |  |  |  |  |  |  |  |  |  |  |  |  |
| April-June | 0.10 | 0.940 |  |  |  |  |  |  |  |  |  |  |  |  |
| April-Sept | 4.08 | **0.001** |  |  |  |  |  |  |  |  |  |  |  |  |
| June-Sept | 5.33 | **0.001** |  |  |  |  |  |  |  |  |  |  |  |  |

P-values for bacteria and AOA obtained from Monte-Carlo test, P (MC) while those for archaea and β-AOB obtained from permutation, P (Perm).
