# Supplementary material for: The Link between Microbial Diversity and Nitrogen Cycling in Marine Sediments Is Modulated by Macrofaunal Bioturbation
Source: PLoS One. 2015 Jun 23;10(6):e0130116. doi: 10.1371/journal.pone.0130116 (PMC4477903; doi:10.1371/journal.pone.0130116)
Supplement: S7 Table — P-values for β-AOB and AOA obtained from Monte-Carlo test, P (MC) while those for bacteria and archaea obtained from permutation, P (Perm). (DOC) [file pone.0130116.s008.doc]

**S7 Table. Pairwise test results from PERMANOVA analysis for temporal differences of microbial Shannon diversity.**

| ***Shannon diversity* Muddy stations** | | | | | | **Fine sandy stations** | | | | | **Permeable stations** | | | |
| --- | --- | --- | --- | --- | --- | --- | --- | --- | --- | --- | --- | --- | --- | --- |
|  | 130 | | 145 | | 700 | | 120 | | 780 | | 230 | | 710 | |
| **β-AOB** | t | P | t | P | t | P | t | P | t | P | t | P | t | P |
|  |  |  |  |  |  |  |  |  |  |  |  |  |  |  |
| April-June | 1.16 | 0.322 | 4.12 | **0.025** | 0.02 | 0.981 | 0.83 | 0.440 | 1.40 | 0.232 | 0.55 | 0.594 | 1.97 | 0.100 |
| April-Sept | 0.35 | 0.761 | 3.36 | **0.037** | 0.08 | 0.932 | 1.27 | 0.275 | 7.44 | **0.004** | 1.86 | 0.138 | 1.51 | 0.223 |
| June-Sept | 0.91 | 0.433 | 1.95 | 0.137 | 0.09 | 0.932 | 3.20 | **0.039** | 6.01 | **0.005** | 0.94 | 0.399 | 17.56 | **0.001** |
|  |  |  |  |  |  |  |  |  |  |  |  |  |  |  |
| **AOA** |  |  |  |  |  |  |  |  |  |  |  |  |  |  |
|  |  |  |  |  |  |  |  |  |  |  |  |  |  |  |
| April-June | 1.57 | 0.180 | 1.13 | 0.381 | 1.13 | 0.304 | 2.00 | 0.130 | 0.05 | 0.969 | 0.60 | 0.565 | 7.12 | **0.004** |
| April-Sept | 1.04 | 0.367 | 1.58 | 0.233 | 1.81 | 0.151 | 2.37 | 0.087 | 2.36 | 0.118 | 1.46 | 0.231 | 2.76 | 0.052 |
| June-Sept | 0.79 | 0.489 | 2.61 | 0.062 | 9.65 | **0.002** | 12.50 | **0.001** | 2.07 | 0.105 | 0.76 | 0.511 | 23.35 | **0.001** |
|  |  |  |  |  |  |  |  |  |  |  |  |  |  |  |
| **Bacteria** | t | P |  |  |  |  |  |  |  |  |  |  |  |  |
| April-June | 5.89 | **0.001** |  |  |  |  |  |  |  |  |  |  |  |  |
| April-Sept | 7.62 | **0.001** |  |  |  |  |  |  |  |  |  |  |  |  |
| June-Sept | 11.27 | **0.001** |  |  |  |  |  |  |  |  |  |  |  |  |
|  |  |  |  |  |  |  |  |  |  |  |  |  |  |  |
| **Archaea** |  |  |  |  |  |  |  |  |  |  |  |  |  |  |
|  |  |  |  |  |  |  |  |  |  |  |  |  |  |  |
| April-June | 1.06 | 0.315 |  |  |  |  |  |  |  |  |  |  |  |  |
| April-Sept | 1.76 | 0.086 |  |  |  |  |  |  |  |  |  |  |  |  |
| June-Sept | 0.86 | 0.388 |  |  |  |  |  |  |  |  |  |  |  |  |

P-values for β-AOB and AOA obtained from Monte-Carlo test, P (MC) while those for bacteria and archaea obtained from permutation, P (Perm).
