# Supplementary material for: The Link between Microbial Diversity and Nitrogen Cycling in Marine Sediments Is Modulated by Macrofaunal Bioturbation
Source: PLoS One. 2015 Jun 23;10(6):e0130116. doi: 10.1371/journal.pone.0130116 (PMC4477903; doi:10.1371/journal.pone.0130116)
Supplement: S8 Table — P-values for bacteria and AOA obtained from Monte-Carlo test, P (MC) while those for archaea and β-AOB obtained from permutation, P (Perm). (DOC) [file pone.0130116.s009.doc]

**S8 Table. Pairwise test results from PERMANOVA analysis for spatial differences of microbial OTU richness.**

| ***OTU richness***  **Muddy stations** | | | | | | | | **Fine sandy stations** | | | | **Permeable stations** | | | |
| --- | --- | --- | --- | --- | --- | --- | --- | --- | --- | --- | --- | --- | --- | --- | --- |
|  | | 130 | | 145 | | 700 | | 120 | | 780 | | 230 | | 710 | |
| **Bacteria** | | t | P | t | P | t | P | t | P | t | P | t | P | t | P |
|  | 130 |  |  |  |  |  |  |  |  |  |  |  |  |  |  |
|  | 145 | 1.97 | 0.121 |  |  |  |  |  |  |  |  |  |  |  |  |
| April | 700 | 0.02 | **0.041** | 0.65 | 0.553 |  |  |  |  |  |  |  |  |  |  |
|  | 120 | 2.30 | 0.084 | 0.14 | 0.895 | 0.87 | 0.440 |  |  |  |  |  |  |  |  |
|  | 780 | 11.00 | **0.000** | 1.86 | 0.135 | 1.06 | 0.343 | 2.50 | 0.066 |  |  |  |  |  |  |
|  | 230 | 1.44 | 0.224 | 0.24 | 0.822 | 0.86 | 0.442 | 0.13 | 0.899 | 1.98 | 0.120 |  |  |  |  |
|  | 710 | 1.73 | 0.160 | 2.40 | 0.072 | 3.41 | **0.025** | 2.77 | 0.053 | 9.45 | **0.000** | 1.85 | 0.137 |  |  |
|  |  |  |  |  |  |  |  |  |  |  |  |  |  |  |  |
|  | 130 |  |  |  |  |  |  |  |  |  |  |  |  |  |  |
|  | 145 | 2.52 | 0.065 |  |  |  |  |  |  |  |  |  |  |  |  |
| June | 700 | 5.50 | **0.005** | 5.33 | **0.007** |  |  |  |  |  |  |  |  |  |  |
|  | 120 | 4.33 | **0.012** | 0.80 | 0.469 | 8.22 | **0.001** |  |  |  |  |  |  |  |  |
|  | 780 | 1.89 | 0.130 | 3.28 | **0.032** | 0.20 | 0.855 | 4.29 | **0.013** |  |  |  |  |  |  |
|  | 230 | 7.35 | **0.001** | 1.61 | 0.180 | 14.50 | **0.000** | 0.86 | 0.436 | 5.30 | **0.006** |  |  |  |  |
|  | 710 | 1.71 | 0.163 | 0.51 | 0.634 | 4.25 | **0.013** | 1.33 | 0.251 | 2.71 | 0.053 | 2.13 | 0.101 |  |  |
|  |  |  |  |  |  |  |  |  |  |  |  |  |  |  |  |
|  | 130 |  |  |  |  |  |  |  |  |  |  |  |  |  |  |
|  | 145 | 0.78 | 0.477 |  |  |  |  |  |  |  |  |  |  |  |  |
| Sept | 700 | 0.00 | 1.000 | 0.76 | 0.488 |  |  |  |  |  |  |  |  |  |  |
|  | 120 | 8.50 | **0.001** | 0.70 | 0.530 | 4.71 | **0.008** |  |  |  |  |  |  |  |  |
|  | 780 | 0.17 | 0.870 | 0.63 | 0.550 | 0.15 | 0.892 | 2.83 | 0.051 |  |  |  |  |  |  |
|  | 230 | 2.29 | 0.080 | 0.08 | 0.940 | 1.89 | 0.129 | 1.70 | 0.162 | 1.31 | 0.255 |  |  |  |  |
|  | 710 | 2.40 | 0.072 | 0.46 | 0.660 | 2.16 | 0.095 | 0.33 | 0.760 | 1.71 | 0.161 | 0.70 | 0.536 |  |  |
|  |  |  |  |  |  |  |  |  |  |  |  |  |  |  |  |
| **AOA** | |  |  |  |  |  |  |  |  |  |  |  |  |  |  |
|  | 130 |  |  |  |  |  |  |  |  |  |  |  |  |  |  |
|  | 145 | 1.03 | 0.358 |  |  |  |  |  |  |  |  |  |  |  |  |
| April | 700 | 2.33 | 0.086 | 0.35 | 0.761 |  |  |  |  |  |  |  |  |  |  |
|  | 120 | 5.92 | **0.004** | 0.10 | 0.438 | 0.84 | 0.435 |  |  |  |  |  |  |  |  |
|  | 780 | 5.83 | **0.005** | 0.85 | 0.478 | 0.68 | 0.530 | 0.26 | 0.835 |  |  |  |  |  |  |
|  | 230 | 1.55 | 0.179 | 0.37 | 0.752 | 0.14 | 0.890 | 0.37 | 0.730 | 0.27 | 0.811 |  |  |  |  |
|  | 710 | 2.06 | 0.119 | 0.07 | 0.950 | 0.43 | 0.680 | 1.51 | 0.220 | 1.34 | 0.240 | 0.44 | 0.652 |  |  |
|  |  |  |  |  |  |  |  |  |  |  |  |  |  |  |  |
|  | 130 |  |  |  |  |  |  |  |  |  |  |  |  |  |  |
|  | 145 | 0.80 | 0.483 |  |  |  |  |  |  |  |  |  |  |  |  |
| June | 700 | 2.68 | 0.052 | 0.43 | 0.679 |  |  |  |  |  |  |  |  |  |  |
|  | 120 | 21.50 | **0.001** | 8.51 | **0.001** | 37.00 | **0.001** |  |  |  |  |  |  |  |  |
|  | 780 | 3.88 | **0.013** | 2.89 | 0.051 | 3.14 | **0.026** | 2.16 | 0.101 |  |  |  |  |  |  |
|  | 230 | 0.86 | 0.438 | 0.57 | 0.559 | 0.46 | 0.684 | 2.00 | 0.126 | 0.91 | 0.433 |  |  |  |  |
|  | 710 | 21.50 | **0.001** | 8.51 | **0.003** | 37.00 | **0.001** | 0.45 | 0.674 | 2.16 | 0.096 | 2.00 | 0.133 |  |  |
|  |  |  |  |  |  |  |  |  |  |  |  |  |  |  |  |
|  | 130 |  |  |  |  |  |  |  |  |  |  |  |  |  |  |
|  | 145 | 1.79 | 0.134 |  |  |  |  |  |  |  |  |  |  |  |  |
| Sept | 700 | 2.79 | **0.046** | 3.62 | **0.028** |  |  |  |  |  |  |  |  |  |  |
|  | 120 | 0.92 | 0.453 | 0.51 | 0.644 | 1.52 | 0.211 |  |  |  |  |  |  |  |  |
|  | 780 | 1.06 | 0.310 | 0.35 | 0.721 | 1.40 | 0.234 | 0.12 | 0.917 |  |  |  |  |  |  |
|  | 230 | 0.60 | 0.618 | 0.90 | 0.423 | 1.87 | 0.141 | 0.30 | 0.790 | 0.43 | 0.701 |  |  |  |  |
|  | 710 | 2.28 | 0.087 | 1.41 | 0.242 | 0.67 | 0.538 | 1.10 | 0.351 | 0.97 | 0.398 | 1.45 | 0.216 |  |  |
| **Archaea** | |  |  |  |  |  |  |  |  |  |  |  |  |  |  |
|  | 130 |  |  |  |  |  |  |  |  |  |  |  |  |  |  |
|  | 145 | 1.86 | 0.082 |  |  |  |  |  |  |  |  |  |  |  |  |
|  | 700 | 2.53 | **0.031** | 2.95 | **0.014** |  |  |  |  |  |  |  |  |  |  |
|  | 120 | 0.79 | 0.458 | 1.98 | 0.068 | 0.25 | 0.792 |  |  |  |  |  |  |  |  |
|  | 780 | 0.13 | 0.885 | 1.37 | 0.212 | 1.30 | 0.206 | 0.71 | 0.479 |  |  |  |  |  |  |
|  | 230 | 0.05 | **0.011** | 3.25 | **0.007** | 0.90 | 0.385 | 0.61 | 0.536 | 1.66 | 0.102 |  |  |  |  |
|  | 710 | 1.93 | 0.079 | 2.84 | **0.025** | 0.86 | 0.417 | 0.80 | 0.431 | 1.58 | 0.160 | 0.45 | 0.666 |  |  |
|  |  |  |  |  |  |  |  |  |  |  |  |  |  |  |  |
| **β-AOB** | |  |  |  |  |  |  |  |  |  |  |  |  |  |  |
|  | 130 |  |  |  |  |  |  |  |  |  |  |  |  |  |  |
|  | 145 | 3.27 | **0.017** |  |  |  |  |  |  |  |  |  |  |  |  |
|  | 700 | 3.35 | **0.011** | 9.70 | **0.001** |  |  |  |  |  |  |  |  |  |  |
|  | 120 | 0.51 | 0.643 | 0.74 | 0.524 | 1.90 | 0.075 |  |  |  |  |  |  |  |  |
|  | 780 | 4.12 | **0.002** | 1.75 | 0.099 | 8.31 | **0.001** | 1.43 | 0.183 |  |  |  |  |  |  |
|  | 230 | 1.89 | 0.081 | 2.68 | **0.027** | 0.91 | 0.369 | 1.90 | 0.085 | 3.34 | **0.014** |  |  |  |  |
|  | 710 | 5.93 | **0.001** | 7.84 | **0.001** | 4.36 | **0.004** | 4.20 | **0.002** | 8.64 | **0.001** | 1.21 | 0.237 |  |  |

P-values for bacteria and AOA obtained from Monte-Carlo test, P (MC) while those for archaea and β-AOB obtained from permutation, P (Perm).
