# Supplementary material for: The Link between Microbial Diversity and Nitrogen Cycling in Marine Sediments Is Modulated by Macrofaunal Bioturbation
Source: PLoS One. 2015 Jun 23;10(6):e0130116. doi: 10.1371/journal.pone.0130116 (PMC4477903; doi:10.1371/journal.pone.0130116)
Supplement: S9 Table — P-values for β-AOB and AOA obtained from Monte-Carlo test, P (MC) while those for bacteria and archaea obtained from permutation, P (Perm). (DOC) [file pone.0130116.s010.doc]

**S9 Table. Pairwise test results from PERMANOVA analysis for spatial differences of microbial Shannon diversity.**

| ***Shannon diversity***  **Muddy stations** | | | | | | | | **Fine sandy stations** | | | | **Permeable stations** | | | |
| --- | --- | --- | --- | --- | --- | --- | --- | --- | --- | --- | --- | --- | --- | --- | --- |
|  | | 130 | | 145 | | 700 | | 120 | | 780 | | 230 | | 710 | |
| **β-AOB** | | t | P | t | P | t | P | t | P | t | P | t | P | t | P |
|  | 130 |  |  |  |  |  |  |  |  |  |  |  |  |  |  |
|  | 145 | 1.05 | 0.364 |  |  |  |  |  |  |  |  |  |  |  |  |
| April | 700 | 0.15 | 0.875 | 1.68 | 0.195 |  |  |  |  |  |  |  |  |  |  |
|  | 120 | 0.70 | 0.502 | 0.67 | 0.566 | 0.68 | 0.541 |  |  |  |  |  |  |  |  |
|  | 780 | 2.01 | 0.125 | 1.53 | 0.235 | 3.10 | **0.037** | 0.95 | 0.409 |  |  |  |  |  |  |
|  | 230 | 1.89 | 0.148 | 1.64 | 0.194 | 1.88 | 0.133 | 0.65 | 0.520 | 2.22 | 0.087 |  |  |  |  |
|  | 710 | 1.36 | 0.237 | 1.22 | 0.326 | 1.34 | 0.240 | 0.31 | 0.790 | 1.67 | 0.172 | 0.38 | 0.724 |  |  |
|  |  |  |  |  |  |  |  |  |  |  |  |  |  |  |  |
|  | 130 |  |  |  |  |  |  |  |  |  |  |  |  |  |  |
|  | 145 | 3.52 | **0.021** |  |  |  |  |  |  |  |  |  |  |  |  |
| June | 700 | 3.08 | **0.047** | 13.40 | **0.001** |  |  |  |  |  |  |  |  |  |  |
|  | 120 | 0.18 | 0.856 | 2.45 | 0.063 | 1.85 | 0.132 |  |  |  |  |  |  |  |  |
|  | 780 | 2.65 | 0.058 | 0.87 | 0.434 | 8.66 | **0.001** | 2.00 | 0.103 |  |  |  |  |  |  |
|  | 230 | 1.11 | 0.369 | 1.30 | 0.241 | 0.92 | 0.400 | 1.08 | 0.337 | 1.28 | 0.292 |  |  |  |  |
|  | 710 | 57.40 | **0.001** | 256. 92 | **0.001** | 115.70 | **0.001** | 35.82 | **0.001** | 103.80 | **0.001** | 2.00 | 0.115 |  |  |
|  |  |  |  |  |  |  |  |  |  |  |  |  |  |  |  |
|  | 130 |  |  |  |  |  |  |  |  |  |  |  |  |  |  |
|  | 145 | 3.54 | **0.028** |  |  |  |  |  |  |  |  |  |  |  |  |
| Sept | 700 | 0.54 | 0.604 | 4.74 | **0.009** |  |  |  |  |  |  |  |  |  |  |
|  | 120 | 3.54 | **0.026** | 1.03 | 0.404 | 4.029 | **0.019** |  |  |  |  |  |  |  |  |
|  | 780 | 5.05 | **0.006** | 1.66 | 0.167 | 6.83 | **0.003** | 0.20 | 0.857 |  |  |  |  |  |  |
|  | 230 | 0.11 | 0.916 | 1.64 | 0.180 | 0.14 | 0.892 | 2.01 | 0.134 | 2.10 | 0.090 |  |  |  |  |
|  | 710 | 0.46 | 0.681 | 1.77 | 0.153 | 0.85 | 0.483 | 2.20 | 0.096 | 2.51 | 0.073 | 0.39 | 0.719 |  |  |
|  |  |  |  |  |  |  |  |  |  |  |  |  |  |  |  |
| **AOA** | |  |  |  |  |  |  |  |  |  |  |  |  |  |  |
|  | 130 |  |  |  |  |  |  |  |  |  |  |  |  |  |  |
|  | 145 | 1.26 | 0.293 |  |  |  |  |  |  |  |  |  |  |  |  |
| April | 700 | 1.41 | 0.231 | 0.14 | 0.890 |  |  |  |  |  |  |  |  |  |  |
|  | 120 | 2.51 | 0.066 | 0.53 | 0.635 | 0.40 | 0.693 |  |  |  |  |  |  |  |  |
|  | 780 | 2.22 | 0.099 | 0.44 | 0.669 | 0.31 | 0.774 | 0.09 | 0.934 |  |  |  |  |  |  |
|  | 230 | 1.74 | 0.171 | 0.52 | 0.663 | 0.47 | 0.639 | 0.18 | 0.856 | 0.24 | 0.822 |  |  |  |  |
|  | 710 | 1.84 | 0.145 | 0.45 | 0.704 | 0.65 | 0.524 | 1.41 | 0.238 | 1.22 | 0.131 | 1.12 | 0.339 |  |  |
|  |  |  |  |  |  |  |  |  |  |  |  |  |  |  |  |
|  | 130 |  |  |  |  |  |  |  |  |  |  |  |  |  |  |
|  | 145 | 0.25 | 0.815 |  |  |  |  |  |  |  |  |  |  |  |  |
| June | 700 | 2.70 | **0.049** | 1.11 | 0.356 |  |  |  |  |  |  |  |  |  |  |
|  | 120 | 189.68 | **0.001** | 22.23 | **0.001** | 66.40 | **0.001** |  |  |  |  |  |  |  |  |
|  | 780 | 2.08 | 0.114 | 2.10 | 0.125 | 1.91 | 0.130 | 1.85 | 0.115 |  |  |  |  |  |  |
|  | 230 | 0.90 | 0.416 | 0.92 | 0.397 | 0.77 | 0.461 | 2.00 | 0.135 | 0.51 | 0.611 |  |  |  |  |
|  | 710 | 189.68 | **0.001** | 22.23 | **0.001** | 66.40 | **0.001** | 2.00 | 0.137 | 1.86 | 0.130 | 2.00 | 0.110 |  |  |
|  |  |  |  |  |  |  |  |  |  |  |  |  |  |  |  |
|  | 130 |  |  |  |  |  |  |  |  |  |  |  |  |  |  |
|  | 145 | 1.59 | 0.191 |  |  |  |  |  |  |  |  |  |  |  |  |
| Sept | 700 | 1.78 | 0.153 | 1.69 | 0.164 |  |  |  |  |  |  |  |  |  |  |
|  | 120 | 0.93 | 0.412 | 1.09 | 0.331 | 1.48 | 0.194 |  |  |  |  |  |  |  |  |
|  | 780 | 0.87 | 0.449 | 0.83 | 0.442 | 1.12 | 0.345 | 0.00 | 0.998 |  |  |  |  |  |  |
|  | 230 | 0.50 | 0.623 | 0.92 | 0.434 | 1.12 | 0.331 | 0.32 | 0.794 | 0.30 | 0.762 |  |  |  |  |
|  | 710 | 1.75 | 0.148 | 0.77 | 0.497 | 0.12 | 0.906 | 1.36 | 0.257 | 1.08 | 0.353 | 1.12 | 0.330 |  |  |
|  |  |  |  |  |  |  |  |  |  |  |  |  |  |  |  |
| **Bacteria** | |  |  |  |  |  |  |  |  |  |  |  |  |  |  |
|  | 130 |  |  |  |  |  |  |  |  |  |  |  |  |  |  |
|  | 145 | 1.25 | 0.216 |  |  |  |  |  |  |  |  |  |  |  |  |
|  | 700 | 1.07 | 0.335 | 1.75 | 0.108 |  |  |  |  |  |  |  |  |  |  |
|  | 120 | 3.70 | **0.003** | 1.10 | 0.302 | 3.51 | **0.004** |  |  |  |  |  |  |  |  |
|  | 780 | 0.38 | 0.739 | 0.70 | 0.487 | 1.07 | 0.280 | 2.04 | 0.069 |  |  |  |  |  |  |
|  | 230 | 4.31 | **0.003** | 0.99 | 0.339 | 3.66 | **0.003** | 0.27 | 0.775 | 2.02 | 0.065 |  |  |  |  |
|  | 710 | 3.49 | **0.011** | 1.36 | 0.206 | 3.52 | **0.006** | 0.45 | 0.694 | 2.22 | 0.065 | 0.72 | 0.474 |  |  |
|  |  |  |  |  |  |  |  |  |  |  |  |  |  |  |  |
| **Archaea** | |  |  |  |  |  |  |  |  |  |  |  |  |  |  |
|  | 130 |  |  |  |  |  |  |  |  |  |  |  |  |  |  |
|  | 145 | 1.46 | 0.172 |  |  |  |  |  |  |  |  |  |  |  |  |
|  | 700 | 3.68 | **0.005** | 2.61 | **0.023** |  |  |  |  |  |  |  |  |  |  |
|  | 120 | 0.72 | 0.490 | 1.66 | 0.128 | 0.70 | 0.506 |  |  |  |  |  |  |  |  |
|  | 780 | 0.77 | 0.439 | 1.71 | 0.108 | 0.84 | 0.457 | 0.03 | 0.973 |  |  |  |  |  |  |
|  | 230 | 3.66 | **0.006** | 2.65 | **0.025** | 0.18 | 0.852 | 0.75 | 0.494 | 0.90 | 0.418 |  |  |  |  |
|  | 710 | 3.04 | **0.019** | 2.82 | **0.016** | 0.93 | 0.350 | 1.12 | 0.331 | 1.27 | 0.228 | 0.82 | 0.451 |  |  |

P-values for β-AOB and AOA obtained from Monte-Carlo test, P (MC) while those for bacteria and archaea obtained from permutation, P (Perm).
