# Supplementary material for: The Link between Microbial Diversity and Nitrogen Cycling in Marine Sediments Is Modulated by Macrofaunal Bioturbation
Source: PLoS One. 2015 Jun 23;10(6):e0130116. doi: 10.1371/journal.pone.0130116 (PMC4477903; doi:10.1371/journal.pone.0130116)
Supplement: S1 Text — (DOC) [file pone.0130116.s011.doc]

# Supplementary Material and Methods

## PCR amplification of total archaeal and bacterial 16S rDNA

The V3 region of the bacterial 16S rDNA gene was amplified for DGGE using the universal bacterial primers F357 and R518 (S1 Table). Amplifications were performed in volumes of 50μl containing 2 μl of target DNA (>20 ng/μl), 6 μl of 10xPCR buffer (containing 15 mM MgCl_2_; GeneAmp, Applied Biosystems, Life Technologies, [Carlsbad, California](http://en.wikipedia.org/wiki/Carlsbad,_California), USA), 0.4 µg/μl BSA, 0.2 mM dNTPs, 0.5 µM of both primers, and 1.25 U AmpliTaq DNA polymerase (1 U/μl) (Applied Biosystems). The ‘touchdown’ PCR conditions were as follows: after an initial denaturation step at 94 °C for 5 min, 20 cycles were performed consisting of three steps: denaturation (94 °C, 1 min), annealing (65 °C, 1 min, decreasing 0.5 °C cycle^-1^ to 55 °C) and extension (72 °C, 1 min) followed by 10 additional cycles in which the annealing temperature was 55 °C. The final elongation step was performed at 72 °C for 30 min to prevent the formation of artificial double bands in subsequent DGGE analysis (Janse *et al*., 2004).

A nested PCR design was used for 16S rDNA amplification of total archaea due to very low yield from direct PCR in some samples (Benlloch *et al*., 2002). In the first step of PCR, the mixture with a volume of 50 μl consisted of 5.55 μl of 10xTop Taq PCR buffer (containing 15 mM MgCl_2_; Qiagen, Hilden, Germany), 0.4 µg/μl of BSA, 0.2 mM of each dNTP, 0.5 µM of primers A8F and A1492R (S1 Table) amplifying approximately 1400 bp of the 16S rDNA genes of archaea, 2.5 U of Top Taq DNA Polymerase (Qiagen) and 5 μl DNA template (>20 ng/μl). The PCR amplification consisted of 5 min at 94 °C, 30 cycles of 1 min denaturation at 94 °C, 90 s annealing at 51 °C, 2 min extension at 72 °C, and a final step at 72 °C for 6 min. PCR products were checked on agarose gel, purified and diluted to 26 ng/μl. In the second step, a ‘touchdown’ PCR was performed for DGGE with primers A344F and A915R to amplify 571 bp of the 16S rDNA fragments in archaea. To prevent complete melting of amplicons during DGGE, a 40-bp-long GC clamp was attached to the 5' end of primer A344F (S1 Table). PCR mixtures with a volume of 25 μl consisted 2.5 μl of 10xCoralLoad, 2.5 μl of 10xTop Taq PCR buffer (containing 15 mM MgCl_2_), 0.005 µg/μl of BSA, 0.2 mM of each dNTP, 0.5 µM of primers A344F and A915R, 0.625 U of Top Taq DNA Polymerase and 1 μl DNA template from the first step. The ‘touchdown’ PCR amplification started with an initial denaturing step at 94 °C for 5 min, followed by 10 cycles with 94 °C for 1 min, 71 °C for 1 min decreasing every cycle by 0.5 °C, and 72 °C for 3 min, followed by another 10 cycles at 94 °C for 1 min, annealing temperature at 61 °C for 1 min, and 72 °C for 3 min. The final elongation step was performed at 72 °C for 30 min.

## cDNA and RT-PCR of archaeal and bacterial *amoA* gene

Integrity and purity of RNA extractions were checked on a 1% agarose gel and by determining the ratio of absorbance at 260 nm and 280 nm and at 260 and 230 nm, which was ≈ 2.0 in all samples. The reverse transcriptase DNase kit (MO BIO Laboratories, Carlsbad, California, USA) was used for the removal of genomic DNA and PCR amplifications with universal primer sets for archaea or bacteria (S1 Table) were performed to verify the complete removal of DNA.

The DNA-free RNA samples were reverse transcribed into cDNA using Omniscript and Sensiscript Reverse Transcriptase Kits (Qiagen) respectively for samples containing ≥ or < 50 ng RNA per reaction according to the instructions of the manufacturer and using 10 µM of Random Hexamer primers (Applied Biosystems) and 2 μl RNA template per total volume of a reaction (20 μl).

RT-PCR amplification of *amoA* gene fragments for DGGE was performed using AOA and β-AOB specific primer sets (S1 Table). RT-PCR mixtures with a volume of 25 μl were the same for AOA and β-AOB regarding the amount of template (5 μl of cDNA) and concentration of all used reagents except for primers and Top Taq DNA Polymerase containing 2.5 μl of 10xCoralLoad, 2.5 μl of 10xTop Taq PCR buffer (containing 15 mM MgCl_2_), 0.005 µg/μl of BSA, 0.2 mM of each dNTP, 0.3 and 0.4 µM of each primer of AOA and β-AOB, respectively, 1.25 and 1.5 U of Top Taq DNA Polymerase for AOA and β-AOB PCR mixtures. The ‘touchdown’ PCR for AOA and β-AOB started with a denaturing step at 94 °C for 5 min. Every cycle consisted of three steps, each for 1 min: 94 °C, annealing temperature and 72 °C. The initial annealing temperature (10 °C higher than annealing temperature, T_an_) was decreased by 0.5 °C per cycle until a touchdown of T_an_. 15 additional cycles were carried out at T_an_ (52.5 °C and 54.5 °C for AOA,s and β-AOB,s primers, respectively) to amplify archaeal and bacterial *amoA* genes. Final primer extension was performed at 72 °C for 30 min.

PCRs were performed in a thermal cycler ([Eppendorf Mastercycler, Hamburg, Germany).](http://eshop.eppendorfna.com/products/Eppendorf_Mastercycler_gradient_personal_thermal_cyclers) All PCR and RT-PCR products were inspected on 1% agarose gels, purified using the Qiaquick PCR purification kit (Qiagen) following the manufacturer’s protocol and measured by NanoDrop 2000 Spectrophotometer (Thermo Scientific, Wilmington, Delaware, USA).

## Denaturing Gradient Gel Electrophoresis (DGGE) of bacteria, archaea, AOA and β-AOB

DGGE analysis of PCR and RT-PCR amplicons was performed using the DCode Universal Mutation Detection System device (Bio-Rad, Hercules, California, USA). To enable fingerprint comparisons across gels, eight prokaryotic DNA samples from previous studies were pooled to generate DGGE standards for gels associated with total bacteria and archaea. Four separate prokaryotic DNA samples were also cloned to use as standards for AOA and β-AOB DGGE gels. Bands in the DGGE standards covered the entire gradient in the gels. 400ng of bacterial and 250ng of archaeal PCR products as well as 350ng of AOA and β-AOB RT-PCR products were loaded depending on the fragment size onto 8% (total bacteria and AOA) and 6% (total archaea and β-AOB) (w/v) polyacrylamide gels (30% [w/v] Bis-Acrylamide solution 37.5:1; National Diagnostics, Charlotte, North Carolina, USA). Optimal electrophoretic separation was obtained using 35-70% (bacteria), 35-65% (archaea), 35-50% (β-AOB) and 30-50% (AOA) denaturing gradients (100% denaturant contains 7 M urea and 40% formamide, Sigma-Aldrich, St. Louis, Missouri, USA), running for 16 h at 75 V in 1xTAE (Tris-acetate-EDTA) buffer at a constant temperature of 60 °C. The gels were stained with SYBR gold (Molecular Probes, Invitrogen, Life Technologies) for 30 min followed by visualization and digital capturing of the profiles via the Molecular Imager Gel Doc XR System (Bio-Rad). Digital images were normalized and processed with BioNumerics (version 5.10, Applied Maths, Sint-Martens-Latem, Belgium).

## Data analysis

The DistLM routine analyses were performed after removing highly correlated independent variables (Draftsmans plot, lrl ≥ 0.90). Fourteen independent variables were entered into the models: chl-*a* concentration, PAP ratio, C:N ratio, MGS (rather than % silt), density of macrofauna, BPc (rather than biomass), diversity and richness of total bacteria, archaea, β-AOB and AOA. Prior to analyses, chl-*a* was ln transformed, whereas density of macrofauna, BPc and PAP ratio were square root transformed to remove right-skewness in the raw data. Predictor variables were then subjected to a sequential step-wise selection procedure using the Akaike’s information selection criterion (AIC) for multivariate response variables and the AIC_c_ criterion in the analysis of univariate response (Anderson *et al*., 2008). To calculate resemblance in Permanova and DistLM, Bray-Curtis similarity and Euclidean distance were used for multivariate (square root transformed) and univariate datasets, respectively.

# Supplementary References

Anderson MJ, Gorley RN, Clarke KR. PERMANOVA + for PRIMER: Guide to software and statistical methods. Plymouth, United Kingdom: PRIMER-E; 2008.

Benlloch S, López-López A, Casamayor EO, Øvreas L, Goddard V, Daae FL, *et al*. Prokaryotic genetic diversity throughout the salinity gradient of a coastal solar saltern. Environ Microbiol. 2002; 4(6): 349–360.

Casamayor EO, Schafer H, Baneras L, Salio CP, Muyzer G. Identification of and spatio-temporal differences between microbial assemblages from two neighbouring sulphurous lakes: comparison by microscopy and denaturing gradient gel electrophoresis. Appl Environ Microbiol. 2000; 66: 499–508.

Hornek R, Pommerening-Roser A, Koops H-P, Farnleitner AH, Kreuzinger N, Kirschner A, Mach RL. Primers containing universal bases reduce multiple *amoA* gene specific DGGE band patterns when analyzing the diversity of beta-ammonia oxidizers in the environment. J Microbiol Meth. 2006; 66: 147–155.

Janse I, Bok J, Zwart G. A simple remedy against artifactual double bands in denaturing gradient gel electrophoresis. J Microbiol Meth. 2004; 57: 279–281.

Muyzer G, De Waal EC, Uitierlinden AG. Profiling of complex microbial populations by Denaturing Gradient Gel Electrophoresis analysis of Polymerase Chain Reaction-amplified Genes Coding for 16S rRNA. Appl Environ Microbiol. 1993; 59(3): 695–700.

Perreault NN, Andersen DT, Pollard WH, Greer CW, Whyte LG. Characterization of the prokaryotic diversity in cold saline perennial springs of the Canadian high arctic. Appl Environ Microbiol. 2007; 73: 1532–1543.

Raskin L, Stromley JM, Rittmann BE, Stahl DA. Groupspecific 16S rRNA hybridization probes to describe natural communities of methanogens. Appl Environ Microbiol. 1994; 60: 1232–1240.

Wuchter C, Abbas B, Coolen MJL, Herfort L, van Bleijswijk J, Timmers P, *et al*. Archaeal nitrification in the ocean. PNAS. 2006; 103(33): 12317–12322.
